# LPS‐Induced Mitochondrial Damage via SLC41A1‐Mediated Magnesium Ion Efflux Leads to the Pyroptosis of Dental Stem Cells

**DOI:** 10.1002/advs.202505666

**Published:** 2025-08-19

**Authors:** Yuan Liu, Chenyu Song, Liyuan Zhang, Xue Han, Chaoyuan Li, Yanhong Yan, Ludan Xing, Mengting Si, Bo Yang, Lingyuan Cheng, Akimi Muramatsu, Beizhan Jiang

**Affiliations:** ^1^ Shanghai Engineering Research Center of Tooth Restoration and Regeneration & Tongji Research Institute of Stomatology & Department of Pediatric Dentistry Shanghai Tongji Stomatological Hospital and Dental School Tongji University Shanghai 200072 China; ^2^ Shanghai Engineering Research Center of Tooth Restoration and Regeneration & Tongji Research Institute of Stomatology & Department of Implantology Shanghai Tongji Stomatological Hospital and Dental School Tongji University Shanghai 200072 China; ^3^ Department of Oral and Maxillofacial‐Head Neck Oncology Ninth People's Hospital Shanghai Jiao Tong University School of Medicine National Clinical Research Center for Oral Diseases Shanghai Key Laboratory of Stomatology Shanghai 200023 China

**Keywords:** dental stem cell, magnesium ion, mitochondrial permeability transition pore, pyroptosis, solute carrier family 41 member 1

## Abstract

Although regenerative endodontics demonstrate promise for dental pulp regeneration, chronic inflammation often hinders the success. This study aims to explore the mechanism whereby lipopolysaccharide (LPS) induces dental pulp regeneration failure. Transcriptomic profiling of LPS‐stimulated dental pulp stem cells (DPSCs) reveals dysregulated cation homeostasis and increased magnesium (Mg^2^⁺) transmembrane transport. Mechanistically, LPS is observed to activate the transcription factor signal transducer and activator of transcription 5A (STAT5A), which binds to the solute carrier family 41 member 1 (*SLC41A1*) promoter, thereby upregulating the Mg^2^⁺ efflux transporter and depleting intracellular Mg^2^⁺ levels. Mg^2^⁺ efflux destabilizes the mitochondrial permeability transition pore (mPTP), thus facilitating its opening via the interaction of oligomycin sensitivity‐conferring protein (OSCP) and cyclophilin D (CypD), which releases reactive oxygen species (ROS) and mitochondrial DNA (mtDNA) and exacerbates oxidative stress. The released mtDNA activates the absent in melanoma 2 (AIM2) inflammasome, thereby amplifying gasdermin D (GSDMD)‐mediated pyroptosis. Exogenous supplementation with Mg^2^⁺ restores intracellular Mg^2^⁺ homeostasis, suppresses mPTP opening, and reduces mtDNA and ROS leakage, thereby rescuing DPSCs viability and differentiation capacity. This study identifies SLC41A1‐mediated Mg^2^⁺ dysregulation as a pivotal driver of LPS‐induced mitochondrial damage and demonstrates that Mg^2^⁺ replenishment is a therapeutic strategy to counteract inflammation‐driven regenerative failure.

## Graphical abstract

Lipopolysaccharide induces upregulation of the magnesium (Mg^2+^) efflux transporter solute carrier family 41 member 1 in dental stem cells, leading to a decrease in intracellular Mg^2+^ concentration and promoting the binding of oligomycin sensitivity‐conferring protein and cyclophilinD. This enhances the sensitivity of mitochondrial permeability transition pore opening and triggers a cascade reaction that affects cell fate.

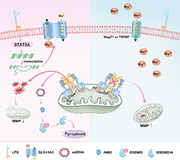

## 1 Introduction

Dental pulp is composed of specialized cells, an extracellular matrix, and neurovascular bundles that sustain tooth vitality by providing essential nutrients and facilitating nociceptive signaling. Despite inherent protective mechanisms, pulp necrosis often occurs after caries or trauma, thus leading to compromised structural integrity and increased risks of tooth fracture. The advancement of regenerative endodontic therapy has made it possible to regenerate intact dental pulp. Although pulp regeneration techniques utilizing dental stem cells (particularly dental pulp stem cells [DPSCs] and stem cells from the apical papilla [SCAP]) have achieved partial success in immature permanent teeth, clinical outcomes remain unpredictable due to persistent inflammatory challenges.^[^ ^1^, ^2^ ^]^ Current limitations stem from an insufficient understanding of how inflammatory microenvironments disrupt regenerative processes at cellular and molecular levels.

Mitochondria are classically recognized as ATP producers via oxidative phosphorylation; however, emerging evidence highlights their role as signaling hubs governing regulated cell death (RCD).^[^ ^3^ ^]^ Mitochondrial integrity depends on coordinated interactions among membrane proteins, enzymes, and matrix components. Lipopolysaccharide (LPS) stimulation induces characteristic ultrastructural pathologies in mitochondria, such as organelle swelling and disrupted membrane integrity.^[^ ^4^ ^]^ These morphological changes trigger the release of mitochondrial damage‐associated molecular patterns (mtDAMPs), mainly including reactive oxygen species (ROS) and mitochondrial DNA (mtDNA).^[^ ^5^, ^6^ ^]^ This release exacerbates mitochondrial damage and activates inflammatory cascades that disrupt cellular homeostasis.^[^ ^7^ ^]^ Emerging evidence links mtDAMPs to key cell death pathways, such as apoptosis, necroptosis, and inflammasome‐mediated pyroptosis.^[^ ^7^ ^]^ For example, cytosolic mtDNA triggers the cGAS‐STING axis, perpetuating a cell death cycle through this signaling cascade.^[^ ^8^, ^9^ ^]^ Additionally, mitochondrial homeostasis plays a decisive role in driving stem cell differentiation. This is exemplified by the loss of pluripotency and impaired differentiation observed in growth factor erv1‐like‐deficient embryonic stem cells, wherein dynamin 1‐like‐mediated excessive mitochondrial fission disrupts fate determination.^[^ ^10^, ^11^ ^]^ Considering the role of mitochondria in energy supply, the regulation of cellular activities, and the determination of stem cell fate, these findings collectively suggest that LPS‐induced regenerative failure may originate from mitochondrial damage‐mediated cellular death.

As the most abundant divalent cation, magnesium (Mg^2+^) regulates over 600 enzymatic reactions and metabolic processes and exhibits unique anti‐inflammatory properties that antagonize Ca^2+^‐mediated inflammatory signaling.^[^ ^12^, ^13^ ^]^ The intracellular Mg^2+^ concentration ([Mg^2+^]_i_) is tightly regulated. Genetic screening of human diseases has resulted in the identification of numerous Mg^2+^‐transport proteins, such as transient receptor potential melastatin type 7 (TRPM7), Mg^2+^ transporter 1 (MagT1), cyclin and CBS domain divalent metal cation transport mediator 3 (CNNM3), solute carrier family 41 member 1 (SLC41A1) and solute carrier family 41 member 2 (SLC41A2).^[^ ^14^ ^]^ Deletion of the TRPM7 transporter causes cell death in B cells due to low [Mg^2+^]_i_, which could be partially rescued by culturing the cells in high‐Mg^2+^‐containing medium.^[^ ^15^ ^]^ Our experiment revealed a decrease in [Mg^2+^]_i_ after LPS stimulation. Although Mg^2+^ is required for normal mitochondrial function, the potential link between disrupted Mg^2+^ homeostasis and mitochondrial damage remains unclear.

In this study, we elucidated a mechanism of LPS‐induced pulp regeneration failure via dysregulation of Mg^2+^ homeostasis. LPS challenge significantly upregulated SLC41A1 in dental stem cells, which triggered a marked depletion of [Mg^2+^]_i_. Mechanistic investigations revealed that Mg^2+^ deficiency facilitated pathological interactions between oligomycin sensitivity‐conferring protein (OSCP) and cyclophilin D (CypD), which are key components of the mitochondrial permeability transition pore (mPTP). This molecular interaction induced mPTP opening, which subsequently caused mitochondrial damage and pyroptosis. Importantly, exogenous Mg^2+^ supplementation effectively counteracted LPS‐induced failure of dental pulp regeneration. These findings elucidate how LPS compromises pulp regeneration and further reveal an Mg^2^⁺ homeostasis‐dependent mechanism that safeguards mitochondrial integrity.

## 2 Results

### 2.1 The Positive Effect of Aerobic Respiration on Pulp Regeneration

**Figure 1:**
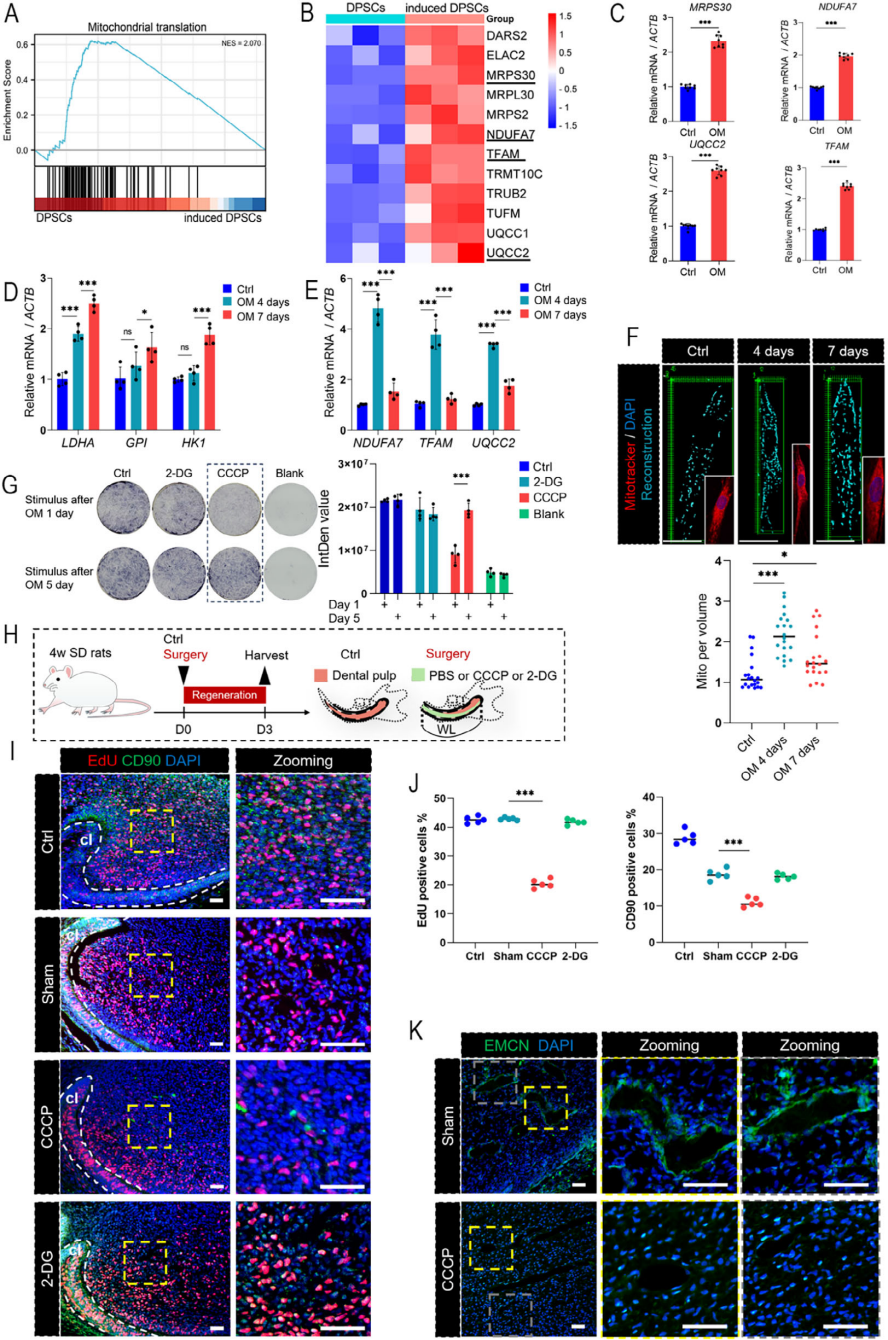
The positive effect of aerobic respiration on pulp regeneration. A) GSEA demonstrating enrichment of mitochondrial translation in DPSCs during neurogenic differentiation. B) Heatmap visualization of the mitochondrial translation‐related gene set GSEA 174 260. C) Following 3 days of induction in osteogenic medium (OM), DPSCs demonstrated significant upregulation of mitochondrial translation‐related genes, including *MRPS30*, *UQCC2*, *NDUFA7*, and *TFAM* (each group *n* = 8). D) The expression of representative genes (*LDHA*, *GPI*, *HK1*) of glycolysis for DPSCs gradually increased during osteogenic induction differentiation, with marked upregulation observed on day 7 (each group *n* = 4). E) The representative genes of aerobic respiration of DPSCs were significantly upregulated at day 4 of osteogenic differentiation, and followed declining by day 7 (each group *n* = 4). F) Mitochondria in DPSCs were labeled with MitoTracker following 4 and 7 days of osteogenic induction, followed by 3D reconstruction. Mitochondrial quantity increased on day 4 but decreased on day 7. Scale bar = 10 µm. G) ALP staining to DPSCs after osteogenic differentiation (each group *n* = 5). Interventions were conducted at day 1 or 5 of osteogenic induction, respectively. The CCCP (10 µм) intervention at day 1 of induction significantly inhibited osteogenic differentiation. The 2‐DG (2 mM) intervention has no significant difference at day 1 or day 5. H) Schematic diagram depicting the construction of rat incisor pulp injury model, with injection of PBS or inhibitors into the root canal. Samples were collected on day 3 after surgery. WL: working length. I) Representative immunofluorescence analysis of rat incisor pulp stem cell niches (each group *n* = 5). EdU labeled proliferating cells and CD90 labeled stem cells. Scale bar = 100 µm. cl: cervical loop. J) Statistical analysis of EdU⁺ and CD90⁺ cells. In the CCCP group, the number of positive cells decreased significantly. K) Representative immunofluorescence analysis was performed on rat incisor pulp using the vascular marker EMCN. The expression of CCCP group was weaker than that of the sham group (each group *n* = 5). Scale bar = 100 µm. ns, no significance; ^*^ *p *< 0.05, ^***^ *p *< 0.001.

Stem cell differentiation requires dynamic regulation of mitochondrial metabolism, with a transition between oxidative phosphorylation and glycolysis required to meet stage‐specific bioenergetic demands.^[^ ^10^ ^]^ To elucidate metabolic interactions during dental pulp regeneration, we analyzed the transcriptomic dataset GSE174260, which captures the differentiation trajectories of DPSCs undergoing neurogenic induction. Gene set enrichment analysis (GSEA) revealed that “mitochondrial translation”‐related genes were upregulated (**Figure** **1**A,B). To simulate the regeneration process of dental pulp, DPSCs and SCAP were isolated, characterized, and subsequently subjected to osteogenic induction for 3 days (Figure S1A–C, Supporting Information). The representative mitochondrial translation genes (mitochondrial ribosomal protein S30 [*MRPS30*], NADH:ubiquinone oxidoreductase subunit A7 [*NDUFA7*], transcription factor A, mitochondrial [*TFAM*] and ubiquinol‐cytochrome c reductase complex assembly factor 2 [*UQCC2*]) from the dataset were upregulated by more than 2‐fold, which confirmed that mitochondrial translation activity was increased (Figure 1C; Figure S1D, Supporting Information). However, fine regulation of energy metabolism via glycolysis is crucial for stem cell differentiation.^[^ ^16^ ^]^ We further analyzed the expression of representative glycolysis‐related genes (including lactate dehydrogenase A [*LDHA*], glucose‐6‐phosphate isomerase [*GPI*] and hexokinase 1 [*HK1*]) during induction. Compared with aerobic respiration, the expression of glycolysis genes was upregulated and more pronounced in the late stage of induction (7 days) (Figure 1D,E; Figure S1E, Supporting Information). To directly quantify changes in the number of mitochondria, we tracked mitochondria via MitoTracker staining. Strikingly, the number of mitochondria increased by day 4 but declined by day 7, which paralleled the transient dominance of oxidative phosphorylation (Figure 1F; Figure S1F, Supporting Information). To elucidate the functional relevance of these metabolic programs, we inhibited aerobic respiration by using carbonyl cyanide m‐chlorophenylhydrazone (CCCP) and glycolysis by using 2‐deoxy‐D‐glucose (2‐DG) during osteogenic induction of DPSCs and SCAP (Figure 1G; Figure S1G, Supporting Information). Early CCCP treatment (stimulus on day 1) significantly reduced osteogenic efficiency, as shown via ALP staining, whereas its impact was diminished postdifferentiation (stimulus on day 5). In contrast, 2‐DG had no significant effect, regardless of intervention timing (day 1 or day 5). These in vitro experiments suggest that early differentiation induction of stem cells requires mitochondria to provide energy.

Due to the multidirectional differentiation potential of stem cell niches at the apical bud, the dental pulp of rat incisors can continuously grow. Under physiological conditions, the differentiation of stem cells can be fully observed from the apical bud to the cut edge. The cells near the apex remain in an undifferentiated state, whereas those cells toward the cut edge gradually differentiate. To simulate pulp regeneration, mandibular incisors of rats were transected at the gingival level, followed by mechanical disruption of pulp tissue using a dental K‐file (working length: 20.7 mm) until reaching the stem cell niche. Histomorphological analyses at 1, 3, and 7 days postsurgery revealed the progressive replacement of the damaged pulp with regenerated pulp tissue (Figures S1I and S2A, Supporting Information). To verify the effects of metabolic pathways on pulp regeneration, we injected CCCP or 2‐DG into the damaged root canal (Figure 1H; Figure S1H, Supporting Information). The expression of the stem cell marker Thy‐1 cell surface antigen (CD90) and the differentiation marker dentin sialophosphoprotein (DSPP) in the CCCP‐treated group was inhibited, and 5‐ethynyl‐2′‐deoxyuridine (EdU) decreased the proportion of proliferating cells by day 3 (Figure 1I,J). However, the 2‐DG group exhibited no significant effect. In addition, compared with the sham treatment, CCCP significantly inhibited the expression of the angiogenic marker Endomucin (EMCN) (Figure 1K). On day 7, despite an increase in EdU⁺ and CD90⁺ cells and elevated EMCN expression, these levels remained lower than those observed in the Sham group (Figure S1J,K, Supporting Information). These results indicate that mitochondria‐mediated aerobic respiration is essential for dental pulp regeneration.

### 2.2 LPS Disrupted Mitochondrial Morphology and Function in Dental Stem Cells

**Figure 2:**
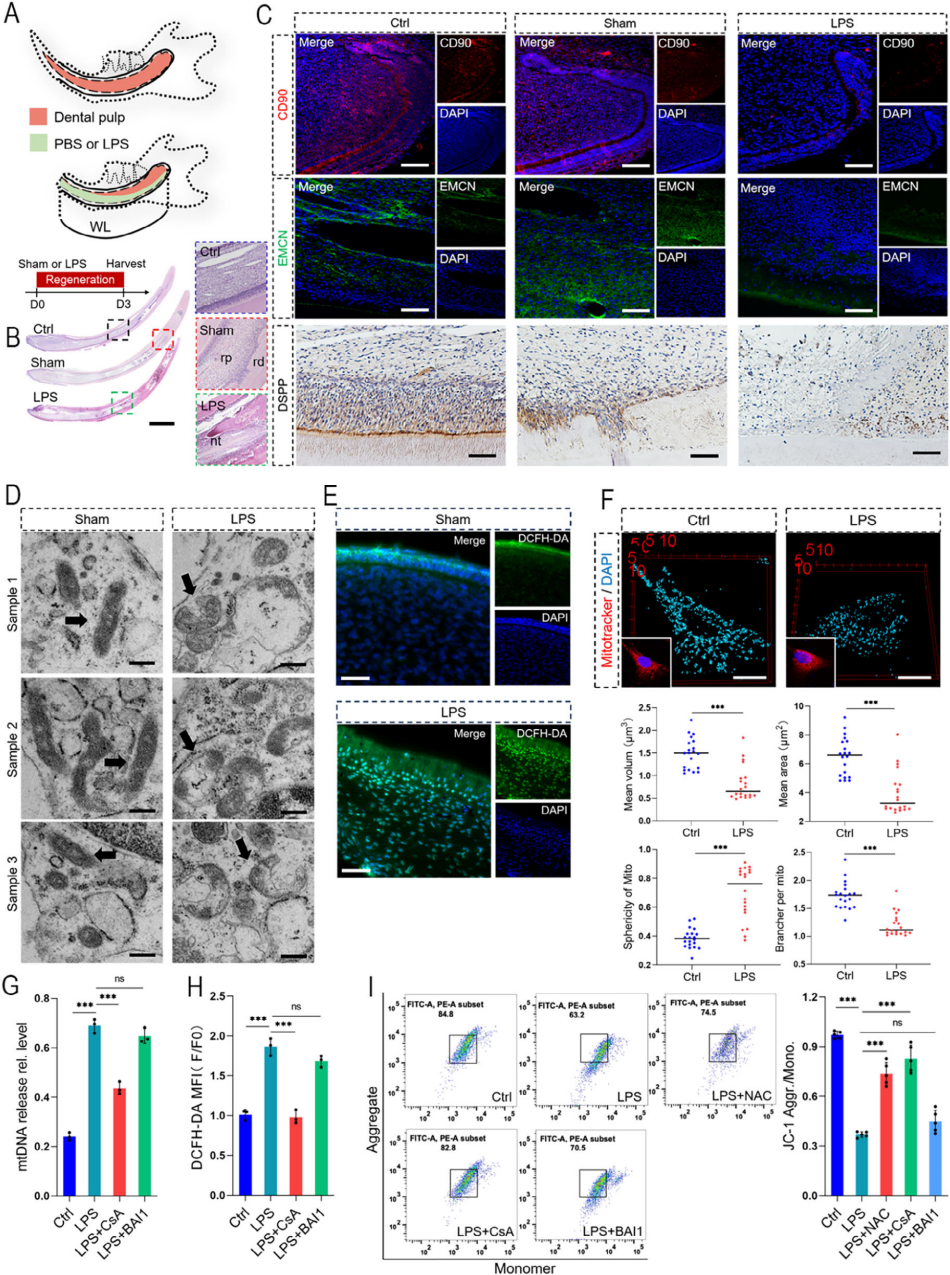
Changes in mitochondria during LPS‐induced pulp regeneration failure. A) Schematic diagram of LPS‐stimulated rat incisor pulp. After damaging the pulp of the rat incisors, LPS was injected into the root canal and sealing. Collect samples at day 3 after surgery. WL: working length. B) HE staining showed necrotic tissue in dental pulp after LPS stimulation (each group *n* = 5). Scale bar = 2 mm. rp: regenerative pulp; rd: regenerative dentin; nt: necrosis tissue. C) Immunofluorescence showed LPS stimulation led to a decrease in the expression of CD90 and EMCN during pulp regeneration. Immunohistochemical analysis also revealed a decrease in DSPP expression (each group *n* = 5). Scale bar = 100 µm. D) Transmission electron microscopy analysis of mitochondrial ultrastructure. LPS stimulation caused mitochondrial swelling and rupture. The black arrow indicated mitochondria (each group *n* = 3). Scale bar = 500 nm. E) Representative immunofluorescence of DCFH‐DA analysis in regenerative dental pulp tissue (each group *n* = 5). Compared to the control group, LPS stimulation caused an increase in ROS. Scale bar = 100 µm. F) In vitro, DPSCs mitochondria were labelled with MitoTracker for mitochondrial morphological analysis. After LPS stimulation, the sphericity increased, indicating mitochondrial expansion from rod‐shaped to spherical, while the average volume, average area, and brancher per mitochondria decreased, suggesting mitochondrial fragmentation. Scale bar = 10 µm. G) Analysis of mtDNA release in DPSCs. LPS stimulation induced mtDNA release, CsA (2 µм) inhibited its release, but BAI1 (500 nM) had a weaker inhibitory effect (each group *n* = 3). H) Analysis of ROS in DPSCs showed that LPS induced an increase in cellular ROS, while CsA (2 µм) could inhibit the increase in ROS. But BAI1 (500 nM) had a weaker inhibitory effect (each group *n* = 3). I) Representative flow cytometry plots of JC‐1 in DPSCs. The ratio of aggregate/monomer represented the level of MMP. LPS stimulation caused a decrease in MMP, while CsA (2 µм) inhibited the decrease, but BAI1 (500 nM) had a weaker inhibitory effect. ROS scavenger NAC (5 mM) could also improve MMP (each group *n* = 5). Quantification shown in right. ns, no significance; ***p < 0.001.

LPS is a representative infectious factor commonly observed in root canal infections.^[^ ^17^ ^]^ To simulate the failure of pulp regeneration, we injected LPS (10 mg mL^−1^) into the root canal after pulp injury (**Figure** **2**A).^[^ ^18^ ^]^ The rats were euthanized after three days, and hematoxylin‐eosin (HE) staining revealed that LPS stimulation caused necrotic tissue in the root canal. Immunohistochemistry revealed that the expression of CD90/DSPP/EMCN decreased in the LPS‐stimulated pulp (Figure 2B,C). Considering the positive role of mitochondria in dental pulp regeneration, transmission electron microscopy analysis was performed on the dental pulp tissue from the apical bud, and the mitochondria in the LPS‐stimulated pulp exhibited swelling and rupture, which are signs of mitochondrial damage (Figure 2D). Additionally, an increase in ROS was observed within the dental pulp (measured via DCFH‐DA), suggesting that mitochondrial damage exacerbated oxidative stress (Figure 2E). To validate these in vivo findings and explore the underlying mechanisms, DPSCs and SCAP were stimulated with different concentrations of LPS in vitro. The viabilities of DPSCs and SCAP were obviously inhibited under LPS stimulation at a concentration of 5 µg mL^−1^ (Figure S2B, Supporting Information). By combining MitoTracker labeling and 3D reconstruction, we observed significant morphological remodeling of the mitochondria in the LPS‐treated DPSCs and SCAP, characterized by reduced average volume, diminished surface area, and decreased number of branches per mitochondrion, which cooccurred with an elevated sphericity index, collectively indicating mitochondrial fragmentation and swelling (Figure 2F; Figure S2C, Supporting Information). With respect to mtDAMPs, LPS stimulation induced a 2‐fold increase in mitochondrial release of mtDNA according to qPCR, and the mean fluorescence intensity (MFI) indicated a 1‐fold increase in cytosolic ROS levels compared to the control in DPSCs and SCAP (Figure 2G,H; Figure S2D,E, Supporting Information). Furthermore, LPS stimulation also induced a decrease in the mitochondrial membrane potential (MMP), which was counteracted via the addition of N‐acetylcysteine (NAC, 5 mM), suggesting that the decrease in the MMP is associated with ROS release (Figure 2I; Figure S2F, Supporting Information). The release of mtDAMPs is usually due to disrupted mPTP or the formation of B‐cell lymphoma 2 antagonists/killers and BCL2‐associated X macropores (BCL‐2/BAX).^[^ ^9^, ^19^, ^20^ ^]^ Using their selective inhibitors Cyclosporin A (CsA, 2 µм) and BAI1 (500 nM), it was observed that CsA could block the release of ROS, decrease of MMP and release of mtDNA in DPSCs and SCAP (Figure 2G–I; Figure S2D–F, Supporting Information). The abovementioned results indicate a close relationship between LPS‐induced pulp regeneration failure and mitochondrial damage; however, further explorations are needed to determine the mechanism involved in the induction of mPTP opening.

### 2.3 Transcriptome Analysis Revealed that Mg^2+^ Homeostasis was Disrupted under LPS Stimulation

**Figure 3:**
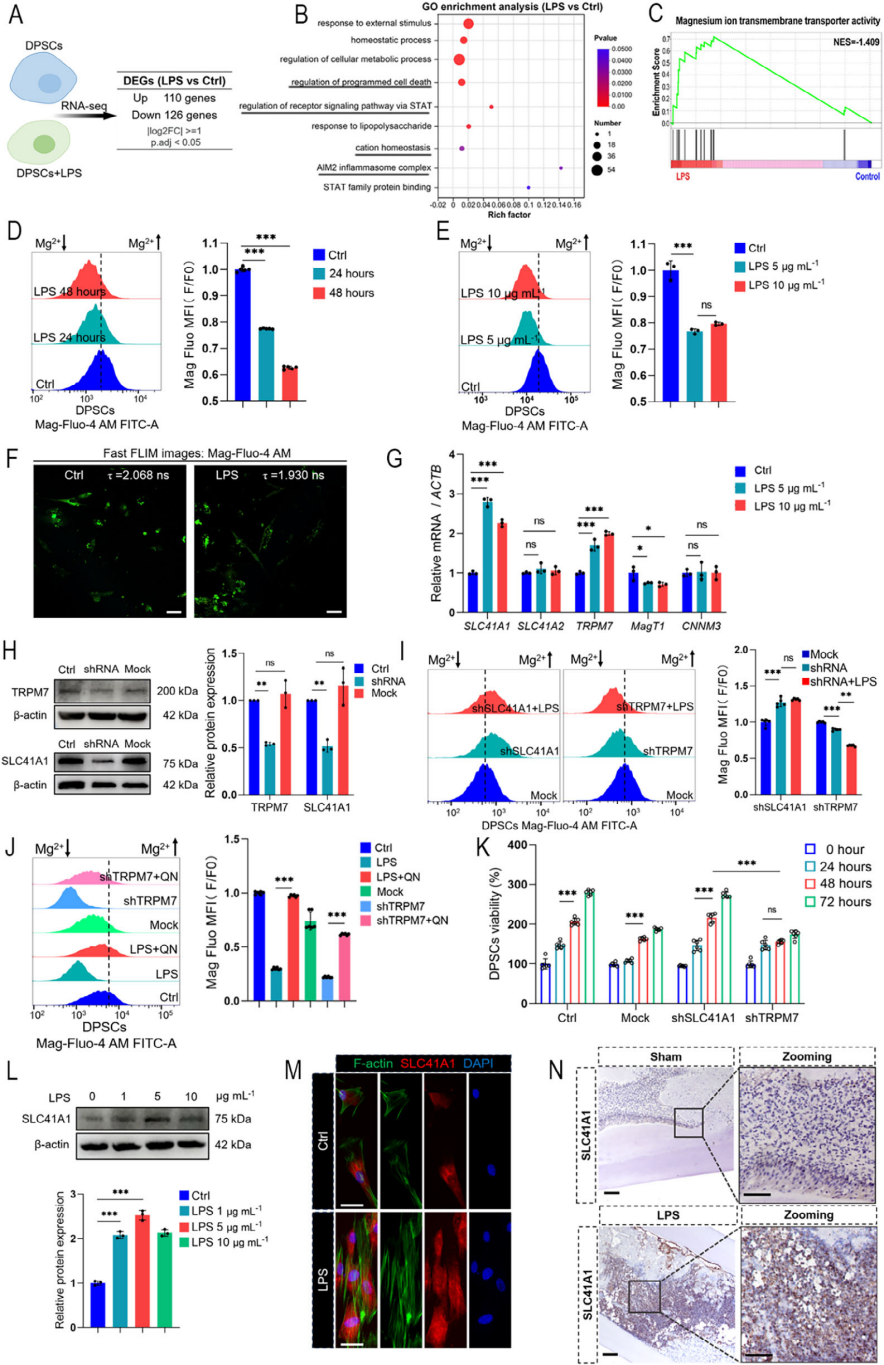
Mechanism exploration of mitochondrial damage in LPS‐induced regeneration failure. A) A schematic diagram of RNA‐seq workflows. B) GO term analysis showing enrichment of cation homeostasis after LPS stimulation. C) GSEA analysis showing enrichment of Mg^2+^ transmembrane transport after LPS stimulation. D) Representative flow cytometry histograms of [Mg^2+^]_i_ (left) and quantification (right). After LPS stimulation of DPSCs, the changes in [Mg^2+^]_i_ were analyzed at 24 and 48 h, and the [Mg^2+^]_i_ gradually decreased (each group *n* = 5). E) Representative flow cytometry histograms of [Mg^2+^]_i_ (left) and quantification (right). There was no difference in the stimulation of DPSCs with concentrations of LPS at 5  and 10 µg mL^−1^ (each group *n* = 3). F) FLIM showing Mg^2^⁺ dynamics following LPS stimulation. Scale bar = 10 µm. G) RT‐qPCR analysis of gene expression of Mg^2+^ transporters. *SLC41A1* and *TRPM7* were significantly upregulated after LPS stimulation to DPSCs (each group *n* = 3). H) Immunoblotting validated the efficiency of TRPM7 and SLC41A1 knockdown in DPSCs (each group *n* = 3). I) Representative flow cytometry histograms of [Mg^2+^]_i_ (left) in DPSCs and quantification (right). Analysis of the effect of knocking down TRPM7 and SLC41A1 on [Mg^2+^]_i_. Knocking down TRPM7 caused a decrease in [Mg^2+^]_i_, which further decreases after LPS stimulation. Knocking down SLC41A1 caused an increase in [Mg^2+^]_i_ (each group *n* = 5). J) Representative flow cytometry histograms of [Mg^2+^]_i_ (left) in DPSCs and quantification (right). Analysis of [Mg^2+^]_i_ after inhibition of SLC41A1 by non‐specific transporter inhibitor quinidine (QN). Both LPS and TRPM7 knockdown caused a decrease in [Mg^2+^]_i_, but quinidine was able to inhibit the decrease in [Mg^2+^]_i_, indicating that quinidine inhibited the sodium/magnesium transporter (each group *n* = 5). K) The effect of knocking down TRPM7 and SLC41A1 on DPSCs viability. Knocking down TRPM7 resulted in a significant decrease in cell viability compared to knocking down SLC41A1, especially after 48 h of cell culture (each group *n* = 5). L) Immunoblotting validated the effect of LPS on SLC41A1 expression in DPSCs. LPS at concentrations of 0, 1, and 5 µg mL^−1^ gradually increased the expression of SLC41A1, but LPS at concentrations of 10 µg mL^−1^ did not further upregulate it (each group *n* = 3). M) Immunofluorescence showed that LPS induced an increase in SLC41A1 expression in DPSCs (each group *n* = 3). Scale bar = 10 µm. N) Immunohistochemical analysis of the rat incisor injury model showed upregulation of SLC41A1 expression in LPS‐induced pulp tissue (each group *n* = 5). Scale bar = 100 µm. ns, no significance; ^**^ *p *< 0.01, ^***^ *p *< 0.001.

To further elucidate the mechanism underlying LPS‐induced mitochondrial damage, transcriptome sequencing was performed on LPS‐stimulated DPSCs, and differentially expressed genes (DEGs) were identified. The Gene Ontology (GO) term highlighted cation homeostasis, and GSEA revealed enrichment of Mg^2^⁺ transmembrane transporter activity (|NES| > 1, *p*‐val < 0.05, FDR *q*‐val < 0.25) (**Figure** **3**A–C). The transcriptome data suggested that Mg^2^⁺ homeostasis may be altered after LPS administration. We performed [Mg^2+^]_i_ analysis in DPSCs and SCAP, as quantified via flow cytometry using an Mg^2^⁺‐sensitive fluorescent probe (Mag‐Fluo‐4 AM). Following 24 h of LPS (5 µg mL^−1^) exposure, the [Mg^2^⁺]_i_ was significantly decreased. This depletion progressed to ≈ 40% upon extending the treatment duration to 48 h, demonstrating a clear time‐dependent pattern (Figure 3D). The same trend was also observed in SCAP (Figure S3A, Supporting Information). However, higher LPS concentrations failed to induce further reduction beyond this level (Figure 3E; Figure S3B, Supporting Information). Critically, fluorescence lifetime imaging microscopy (FLIM) provided deeper insights. The lifetime of the Mg^2^⁺ probe was significantly decreased in the LPS‐treated cells (Figure 3F; Figure S3D, Supporting Information). To elucidate this phenomenon, we hypothesized that the [Mg^2^⁺]_i_ decrease could stem from increased efflux (via transporters such as SLC41A1 and SLC41A2) or reduced influx (via transporters such as TRPM7, MagT1, and CNNM3).^[^ ^13^ ^]^ Representative Mg^2+^ transporter gene expression was detected via RT‐qPCR after LPS stimulation, and *SLC41A1* and *TRPM7* were significantly upregulated in DPSCs and SCAP (Figure 3G; Figure S3C, Supporting Information). In a rat model of incisor injury, immunohistochemical analysis of dental pulp tissue revealed increased SLC41A1 and TRPM7 expression in the LPS‐stimulated group (Figure 3N; Figure S3E, Supporting Information). For further screening, we assessed the effects of the two transporters on [Mg^2+^]_i_ from a functional perspective. SLC41A1 and TRPM7 were knocked down in DPSCs and SCAP, and the results demonstrated that the silencing of SLC41A1 could alleviate the Mg^2+^ efflux caused by LPS, whereas TRPM7 knockdown exacerbated the decrease in [Mg^2^⁺]_i_ (Figure 3H,I; Figure S3F–I, Supporting Information). These results suggest that influx and efflux transporters influence Mg^2+^ homeostasis. Martin et al. reported that quinidine could inhibit SLC41A1‐mediated Mg^2+^ efflux.^[^ ^21^ ^]^ Quinidine rescued [Mg^2^⁺]_i_ levels in LPS‐treated or TRPM7‐silenced DPSCs and SCAP (Figure 3J; Figure S3J, Supporting Information). Additionally, the knockdown of SLC41A1 had no significant effect on cell viability, whereas knockdown of TRPM7 reduced cell viability, thereby indicating that a decrease in [Mg^2+^]_i_ could affect DPSCs and SCAP viability (Figure 3K; Figure S3K, Supporting Information). The abovementioned results indicated that the upregulation of SLC41A1 was the main factor causing Mg^2+^ efflux. Further analysis revealed that LPS (5 µg mL^−1^) upregulated SLC41A1 expression in DPSCs and SCAP, with no additional increase being observed at higher concentrations (Figure 3L; Figure S3L, Supporting Information). Immunofluorescence analysis also revealed that LPS (5 µg mL^−1^) triggered an increase in SLC41A1 expression in DPSCs and SCAP (Figure 3M; Figure S3M, Supporting Information). Collectively, these results demonstrate that LPS primarily disrupts Mg^2^⁺ homeostasis via SLC41A1‐mediated efflux, thereby impairing cell viability.

### 2.4 Overexpression of SLC41A1 Caused Mitochondrial Damage

**Figure 4:**
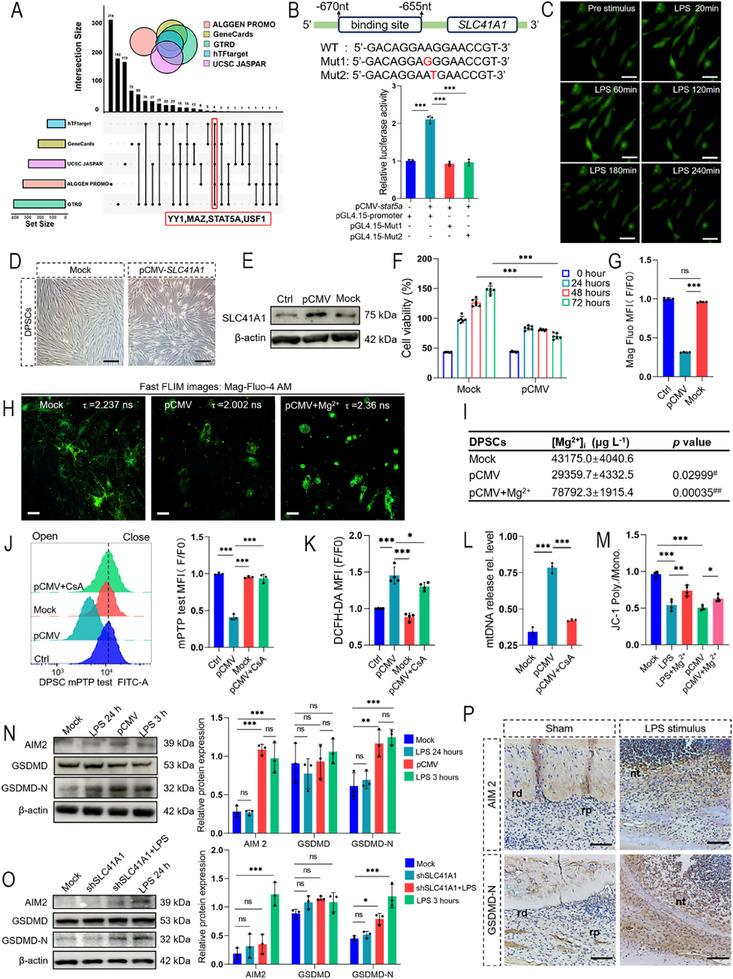
Overexpression of SLC41A1 caused mitochondrial damage in DPSCs. A) Analyze potential transcription factors of *SLC41A1* using 5 databases. Upset Venn plot showed that YY1, MAZ, STAT5A, and USF1 were all predicted as transcription factors. B) The dual luciferase gene reporter assay confirmed that STAT5A was a transcription factor of *SLC41A1*. STAT5A cannot bind to the *SLC41A1* promoter mutant. C) Ca^2+^ probe analysis of changes in cytoplasmic Ca^2+^ in DPSCs. After LPS stimulation of DPSCs, the fluorescence intensity of Ca^2+^ probes increased within 120 min, then began to decrease, and basically returned to normal after 240 min. Scale bar = 25 µm. D) Morphological changes of DPSCs overexpressing SLC41A1 under bright field. After transfection for 24 h, the cell morphology expanded and the edges became bright. Scale bar = 25 µm. E) Immunoblotting validation of SLC41A1 overexpression in DPSCs (pCMV, 24 h). F) Overexpression of SLC41A1 (pCMV) resulted in a significant decrease in DPSCs cell viability after 48 h (each group *n* = 6). G) Overexpression of SLC41A1 (pCMV) led to a decrease in intracellular [Mg^2+^]_i_ after 24 h (each group *n* = 5). H) FLIM revealed shortened fluorescence lifetime post‐transfection in DPSCs, which was prolonged upon Mg^2^⁺ supplementation. Scale bar = 10 µm. I) ICP‐MS detected decreased [Mg^2+^]_i_ levels in DPSCs following overexpression, which increased after Mg^2^⁺ supplementation (each group *n* = 3; #: pCMV versus Mock; ##: pCMV + Mg^2^⁺ versus Mock). J) Representative flow cytometry histograms of mPTP opening (left) and quantification (right). Overexpression of SLC41A1 (pCMV) increased the opening of mPTP in the mitochondrial inner membrane of DPSCs after 24 h (each group *n* = 3). K) Overexpression of SLC41A1 (pCMV) led to an increase in ROS in DPSCs after 24 h (each group *n* = 4). L) Overexpression of SLC41A1 (pCMV) increased mtDNA release in DPSCs after 24 h (each group *n* = 3). M) Overexpression of SLC41A1 (pCMV) led to a decrease in MMP tested by flow cytometry in DPSCs after 24 h (each group *n* = 4). N) Immunoblotting was used to detect the expression of pyroptosis protein in cells overexpressing SLC41A1 (pCMV, 24 h). LPS stimulation after 24 h and overexpression of SLC41A1 both cause upregulation of AIM2 and GSDMD‐N. The LPS (5 µg mL^−1^, 3 h)‐primed and ATP (5 mM, 1 hour)‐stimulated group was the positive control (each group *n* = 3). O) Immunoblotting was used to validate the expression of pyroptosis after knocking down SLC41A1. After knocking down SLC41A1, the expression of AIM2 and GSDMD‐N decreased. P) Upregulation of AIM2 and GSDMD‐N expression in LPS‐induced rat incisor pulp tissue (each group *n* = 3). rp: regenerative pulp; rd: regenerative dentin; nt: necrosis tissue. Scale bar = 100 µm. ns, no significance; ^*^ *p *< 0.05, ^**^ *p *< 0.01, ^***^ *p *< 0.001.

The SLC41A1 gene is located at chromosomal locus 1q32.1. To investigate how LPS induced SLC41A1 upregulation, we integrated predictions from five transcription factor databases (hTFtarget, GeneCards, UCSC JASPAR, ALGGEN PROMO, and GTRD) and identified STAT5A, YY1, USF1, and MAZ as potential transcription factors of the SLC41A1 promoter (**Figure** **4**A). In support of this prediction, the GO term from RNA‐seq indicated enrichment of STAT protein‐related activity, thus prompting us to prioritize STAT5A for mechanistic validation (Figure 3B). To verify its regulatory role, wild‐type and mutant *SLC41A1* promoter luciferase reporter assays confirmed that STAT5A specifically binds to the promoter sequence 5′‐GACAGGAAGGAACCGT‐3′ (located at nucleotide ‐670 ∼ ‐655), thereby establishing its role in transcriptional activation (Figure 4B).

LPS stimulation can trigger RCD via intense calcium (Ca^2^⁺) oscillations.^[^ ^22^ ^]^ Based on live‐cell imaging, LPS was able to induce Ca^2^⁺ flux dynamics (peaking at 2 h posttreatment) in DPSCs followed by normalization, as evidenced by Ca^2^⁺ probe staining analysis (Figure 4C). In contrast, [Mg^2^⁺]_i_ exhibited sustained depletion, implying divergent regulatory pathways for Ca^2^⁺ and Mg^2^⁺ during RCD (Figure 3D; Figure S3A, Supporting Information). To further clarify the mechanism of mitochondrial damage, SLC41A1 was overexpressed in DPSCs and SCAP, with SLC41A1 overexpression leading to morphological abnormalities, thus indicating the occurrence of cell death (Figure 4D–F; Figure S4A–C, Supporting Information). MFI of Mag‐Fluo‐4 AM probe via flow cytometric analysis demonstrated a > 50% decrease in [Mg^2^⁺]_i_ (Figure 4G; Figure S4D, Supporting Information). FLIM provides validation in DPSCs and SCAP (Figure 4H; Figure S4E, Supporting Information). Furthermore, the overexpression of the Mg^2^⁺ channel triggered an over‐efflux of Mg^2^⁺, which was directly quantified via inductively coupled plasma‐mass spectrometry (ICP‐MS), which revealed a significant reduction of [Mg^2^⁺]_i_ in DPSCs and SCAP (Figure 4I; Figure S4F, Supporting Information). Given the involvement of LPS‐induced mitochondrial damage, mPTP status was further investigated. SLC41A1 overexpression triggered mPTP opening, as demonstrated by a 50% decrease in the relative MFI compared to the control. The overexpression also increased the release of ROS and mtDNA (Figure 4J–L; Figure S4G–I, Supporting Information). The MMP was diminished under ROS‐mediated oxidative stress (Figure 4M; Figure S4J, Supporting Information), thus confirming mitochondrial dysfunction.

Regarding the pathway of LPS‐induced RCD in DPSCs, GO term analysis revealed enrichment of the AIM2 inflammasome complex, a pathway responsive to cytosolic mtDNA (Figure 3B).^[^ ^23^ ^]^ Consistent with these findings, immunoblotting confirmed that AIM2 expression was increased and that gasdermin D (GSDMD) was cleaved into GSDMD‐N, which is a pyroptosis‐executing fragment in SLC41A1‐overexpressing DPSCs and SCAP (Figure 4N; Figure S4K, Supporting Information). To establish causality, SLC41A1 knockdown resulted in a lack of upregulation of AIM2 and GSDMD‐N (Figure 4O; Figure S4L, Supporting Information). In vivo, AIM2 and GSDMD‐N were also highly expressed in the LPS‐stimulated dental pulp (Figure 4P). Collectively, these results demonstrate that LPS‐driven SLC41A1 upregulation depletes [Mg^2^⁺]_i_, leading to mPTP destabilization and mtDNA release, which activates the AIM2 inflammasome to execute pyroptosis, ultimately constituting a critical barrier to pulp regeneration.

**Figure 5:**
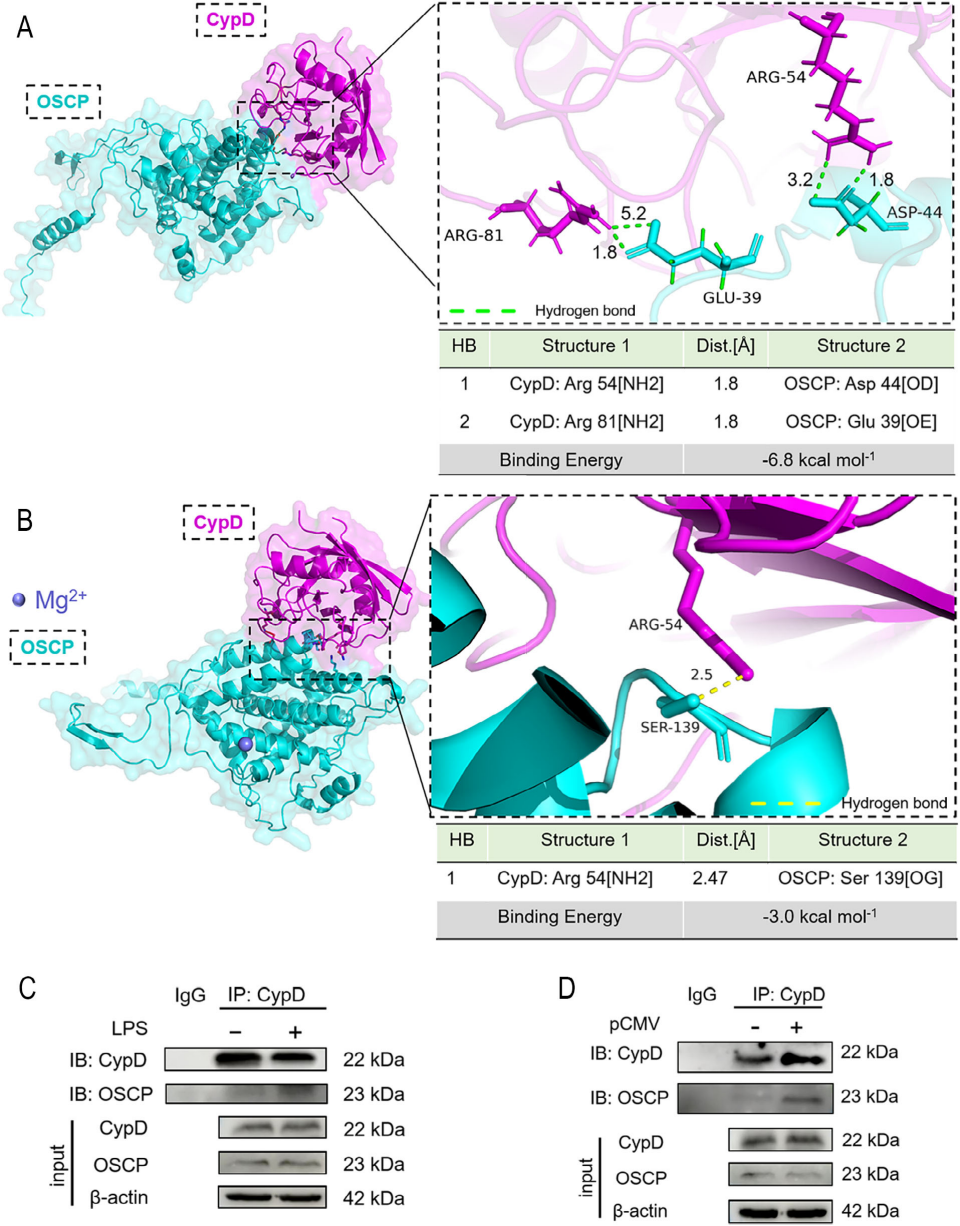
Mg^2+^ could reduce the binding ability between CypD and OSCP. A) Alphafold3 simulated the binding of CypD and OSCP, with a binding energy of −6.8 kcal mol^−1^. Hydrogen bond distances less than 3.0 Å are considered effective binding. B) Alphafold3 simulated the binding of CypD with OSCP‐Mg^2+^, with a binding energy of −3.0 kcal mol^−1^. C) Co‐IP showed that the binding ability between CypD and OSCP was enhanced after LPS stimulation. D) Co‐IP confirmed that overexpression of SLC41A1 (pCMV, 24 h) enhanced the binding between CypD and OSCP.

Notably, CsA suppressed the mtDAMPs release induced by SLC41A1 overexpression, suggesting the involvement of CypD in this process (Figure 4H–J; Figure S4E–G, Supporting Information). Prior studies have demonstrated that CypD can bind to OSCP, thereby increasing the sensitivity of mPTP opening.^[^ ^24^ ^]^ Moreover, Zhang et al. copackaged Mg^2+^ and CypD‐targeting siRNA into a complex delivered to the brain, demonstrating that mPTP modulation could alleviate Alzheimer's disease.^[^ ^25^ ^]^ Previous research has suggested that Mg^2+^ independently regulates the opening of mPTP and that low [Mg^2+^]_i_ causes reversible opening of mPTP.^[^ ^26^ ^]^ However, the mechanism by which Mg^2+^ independently regulates the opening and closing of mPTP has not been further explored. We hypothesized that the presence of Mg^2+^ could hinder the binding of OSCP to CypD, thereby reducing the sensitivity of mPTP opening. Molecular docking revealed that OSCP enables binding with CypD, with a binding energy of −6.8 kcal mol^−1^. A close‐up view of a putative interaction of hydrogen bonds between residues Arg‐54 and Arg‐81 in CypD with residues Asp‐44 and Glu‐39 in OSCP is shown in **Figure** **5**A. Considering that OSCP is a subunit of F1F0‐ATPase and that Mg^2+^ is a key cofactor for F1F0‐ATPase function, it is reasonable to assume that Mg^2+^ binds to OSCP. When Mg^2+^ was bound to the OSCP protein, its binding energy with CypD increased to −3.0 kcal mol^−1^, indicating a decreased binding ability. Only one hydrogen bond existed between Arg‐54 in CypD and Ser‐193 in OSCP‐Mg^2+^ (Figure 5B). Experimental validation via co‐immunoprecipitation (Co‐IP) revealed enhanced CypD‐OSCP binding under both LPS stimulation (Figure 5C) and SLC41A1 overexpression (Figure 5D), consistent with Mg^2^⁺ depletion facilitating mPTP sensitization. Together, these results mechanistically link Mg^2^⁺ homeostasis to the structural dynamics of mPTP regulation.

### 2.5 Exogenous Mg^2+^ Supplementation Alleviated the Mitochondrial Damage Caused by LPS

**Figure 6:**
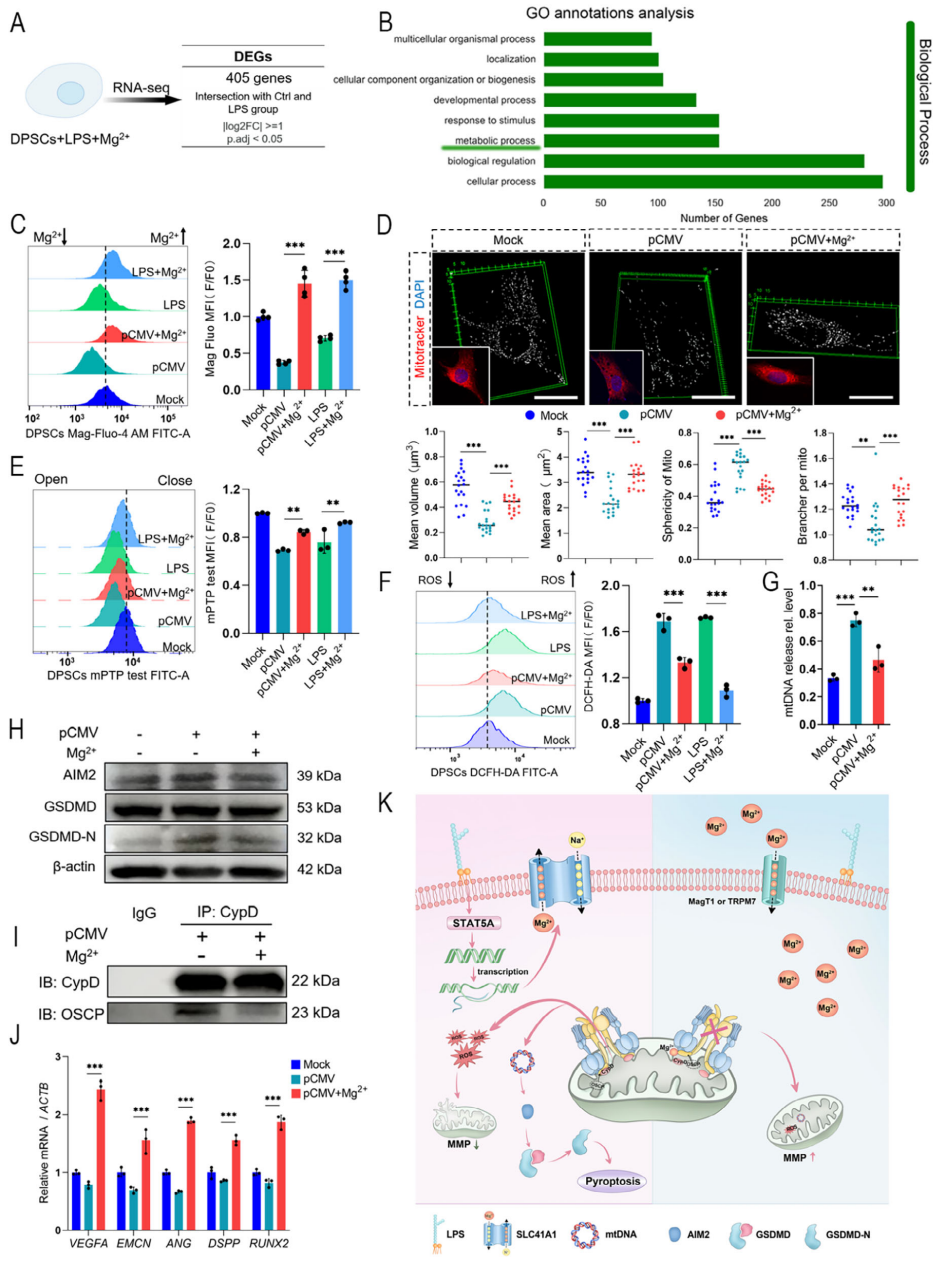
Exogenous Mg^2+^ supplementation alleviated LPS‐induced mitochondrial damage. A) A schematic diagram of RNA‐seq workflows. B) GO term analysis showing enrichment in the metabolic processes of the LPS + Mg^2+^ group. C) Representative flow cytometry histograms of [Mg^2+^]_i_ (left) and quantification (right). Exogenous Mg^2+^ supplement led to an increase in [Mg^2+^]_i_, resisting the Mg^2+^ efflux caused by the upregulation of SLC41A1 (each group *n* = 4). D) In vitro, mitochondria were labelled by MitoTracker and performed with mitochondrial morphological analysis. Overexpression of SLC41A1 (pCMV) lead to an increase in sphericity, indicating mitochondrial expansion from rod‐shaped to spherical, while the average volume, average area, and brancher per mitochondria decreased, suggesting mitochondrial fragmentation. After exogenous supplementation of Mg^2+^, the sphericity decreased, while the average volume, average area, and brancher per mitochondria increased (each group *n* = 20). Scale bar = 10 µm. E) Representative flow cytometry histograms of mPTP opening (left) and quantification (right). Exogenous Mg^2+^ could reduce the mPTP open state caused by LPS (24 h) or overexpression of SLC41A1 (24 h) (each group *n* = 3). F) Representative flow cytometry histograms of ROS test (left) and quantification (right). Exogenous Mg^2+^ could reduce the elevated levels of cytoplasmic ROS caused by LPS (24 h) or overexpression of SLC41A1 (pCMV, 24 h) (each group *n* = 3). G) Exogenous Mg^2+^ could reduce the release of mtDNA caused by LPS (24 h) or overexpression of SLC41A1 (pCMV, 24 h) (each group *n* = 3). H) Exogenous Mg^2+^ downregulated the upregulation of AIM2 and GSDMD‐N caused by overexpression of SLC41A1 (pCMV, 24 h). I) Exogenous Mg^2+^ supplementation inhibited the binding of CypD and OSCP caused by overexpression of SLC41A1 (pCMV, 24 h). J) After inducing vascular or dentinogenic differentiation of DPSCs for 7 days, overexpression of SLC41A1 (pCMV, 24 h) resulted in downregulation of vascular and dentinogenic differentiation genes. Exogenous Mg^2+^ supplement was able to reverse this state (each group *n* = 3). ^**^ *p *< 0.01, ^***^ *p *< 0.001. K) The mechanism diagram of LPS mediated Mg^2+^ homeostasis imbalance causing mitochondrial damage.

Considering the reversibility of mPTP opening and closing and its central role in pathological reactions caused by low [Mg^2^⁺]_i_, we further investigated whether exogenous Mg^2+^ supplementation could alleviate LPS‐induced dental stem cell damage. The DPSCs and SCAP were treated with varying concentrations of magnesium chloride (MgCl_2_). We then analyzed cell viability, mPTP opening, and mtDNA release. Notably, treatment with 5 mM MgCl_2_ significantly restored cell viability and inhibited mPTP opening and mtDNA release (Figure S5A–F, Supporting Information). Afterward, transcriptomic profiling of Mg^2^⁺‐supplemented LPS‐treated DPSCs revealed 405 DEGs, with GO analysis highlighting enrichment in metabolic pathways (**Figure** **6**A,B). To clarify the mechanism of action, we tested [Mg^2+^]_i_ after supplementation. Exogenous Mg^2^⁺ supplementation restored [Mg^2+^]_i_ in DPSCs and SCAP to near physiological levels (control group) (Figure 6C; Figure S5G, Supporting Information). To determine whether Mg^2+^ alleviated mitochondrial damage, we utilized MitoTracker to label mitochondrial morphology. After exogenous supplementation, the average volume, average surface area, and number of branches per mitochondrion were increased, whereas the sphericity was decreased, which was more similar to the physiological status (Figure 6D; Figure S5H, Supporting Information). Mechanistically, the increased MFI suggested that opening of the mPTP was reversed after Mg^2+^ supplementation (Figure 6E; Figure S5I, Supporting Information). Consequently, the cytosolic ROS and mtDNA levels decreased (Figure 6F,G; Figure S5J,K; Supporting Information). Furthermore, the MMP increased due to lower ROS after Mg^2+^ supplementation (Figure 4M; Figure S4J, Supporting Information). For pyroptosis, Mg^2+^ supplementation also downregulated AIM2 and decreased GSDMD cleavage (Figure 6H; Figure S5L, Supporting Information). The binding between CypD and OSCP detected by Co‐IP was also weakened after exogenous Mg^2+^ supplementation (Figure 6I). With the reversal of mitochondrial function, the viability of stem cells improved, and dentinogenesis‐ and angiogenesis differentiation‐related genes were also upregulated by ≈ 2‐fold (Figure 6J; Figure S5M, Supporting Information). In summary, low [Mg^2+^]_i_ causes opening of mPTP, leading to mitochondrial damage and pyroptosis, whereas supplementation with Mg^2+^ can close mPTP, thereby reversing mitochondrial damage and pyroptosis (Figure 6K).

### 2.6 In Vivo Experiments on the Ability of Mg^2+^ to Alleviate the LPS‐Induced Inhibition of Dental Pulp Regeneration

**Figure 7:**
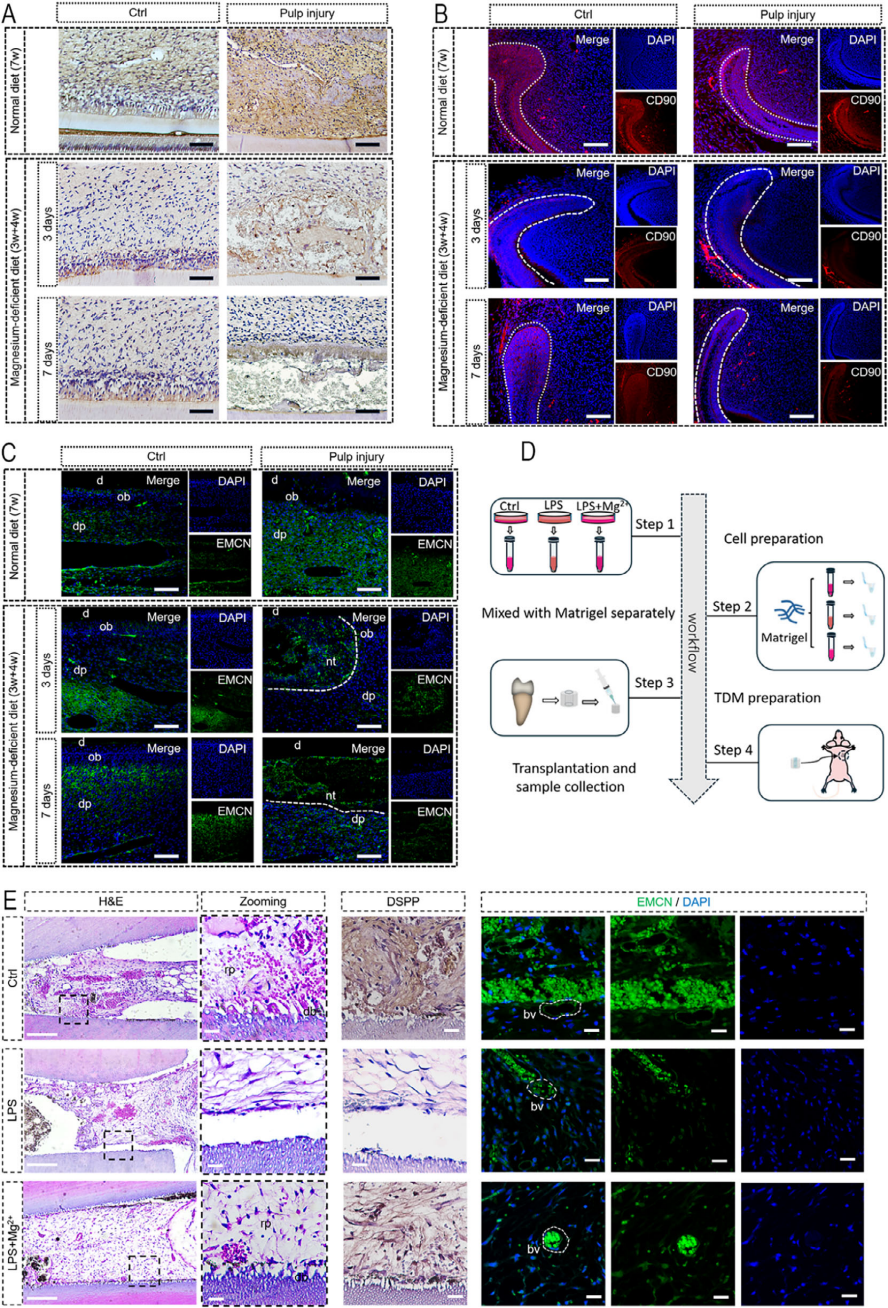
In vivo experimental verification of the effect of Mg^2+^ on pulp regeneration. A) A model of rat incisor pulp injury was established in Mg^2+^‐deficient rats, and compared with normally fed rats, the DSPP expression was reduced. In addition, the DSPP in the regenerative pulp tissue of rats with normal feeding after pulp injury increased, but there was no significant upregulation of DSPP in Mg^2+^‐deficient rats, and there was a large amount of necrotic tissue in the pulp (each group *n* = 5). Scale bar = 100 µm. B) Both the control group and the sham surgery group of rats with normal feeding showed CD90 expression in the dental pulp tissue, but the CD90 reduced in Mg^2+^‐deficient rats compared to normal feeding rats (each group n = 5). Scale bar = 100 µm. C) Compared with normal fed rats, the EMCN expression in Mg^2+^‐deficient rats is similar to that in normal rats, with expression in dental pulp tissue and increased expression near blood vessels. However, after damaging the pulp of the incisors, the EMCN in the regenerative pulp decreased (each group *n* = 5). Scale bar = 100 µm. d: dentin; ob: odontoblast; dp: dental pulp; nt: necrosis tissue. D) Schematic diagram of constructing TDM subcutaneous transplantation model in nude mouse. Scale bar = 1 mm. E) Tissue analysis of TDM model (each group *n* = 5). HE staining analysis showed loose connective tissue in the LPS group, with no cellular connection established with TDM. Immunohistochemistry staining showed almost no expression of DSPP. The LPS + Mg^2+^ group exhibited a filled connective tissue structure, with cells establishing connections with TDM and the presence of newly formed dentinal tubule‐like structures. DSPP also shows an increase in expression. bv: blood vessel. Scale bar = 100 µm.

To validate low [Mg^2+^]_i_‐induced regeneration failure in vivo, we constructed an Mg^2+^‐deficient rat model. Three‐week‐old rats were fed an Mg^2+^‐free diet for four weeks, which was confirmed via characteristic skin lesions (Figure S6A, Supporting Information).^[^ ^26^ ^]^ The Mg^2+^‐deficient group (pulp injury without LPS) demonstrated decreased expression of DSPP (**Figure** **7**A), CD90 (Figure 7B) and EMCN (Figure 7C). These findings indicated that the regenerative ability of incisor pulp in Mg^2+^‐deficient rats was impaired. Immunohistochemistry revealed elevated AIM2 and GSDMD‐N expression in the dental pulp tissues of Mg^2^⁺‐deficient rats, particularly in the predentin‐adjacent regions (Figure S6B,C, Supporting Information). The incisor pulp was subsequently destroyed (without LPS stimulation), and the pulp exhibited extensive necrotic areas, with pronounced AIM2 and GSDMD‐N immunoreactivity localized to the perinecrotic zones (Figure S6B,C, Supporting Information). These findings indicate that pyroptosis actively occurs in Mg^2^⁺‐deficient dental pulp.

To simulate the dental root environment and stimulate tissue regeneration, a treated dentin matrix (TDM) model was applied via subcutaneous transplantation.^[^ ^27^ ^]^ P2 DPSCs treated with LPS or LPS + Mg^2+^ were mixed with Matrigel and inserted into TDM before subcutaneous transplantation into immunocompromised mice for 4 weeks (Figure 7D). The normal human dental pulp tissue involved sparse connective tissue filling the pulp cavity, with visible vascular structures and relatively high expression of DSPP being detected near the dentin (Figure S6D, Supporting Information). The Matrigel vehicle group exhibited connective tissue formation within the TDM, characterized by a predominance of fibrous elements over cellular elements, absence of DSPP expression, and obliteration of structural demarcation between the newly formed tissue and the peripheral encapsulation layer (Figure S6D, Supporting Information). In the control group (DPSCs + Matrigel), pulp‐like tissue formed; however, the tissue could not fully fill the tooth segment (Figure 7E). Following LPS challenge (DPSCs + 5 µg mL^−1^ LPS + Matrigel), complete failure of cellular adhesion to dentin and low DSPP expression were observed. After LPS + Mg^2+^ intervention (DPSCs + 5 µg mL^−1^ LPS + 5 mM Mg^2+^ + Matrigel), the tissue became filled with TDM and resembled dental pulp (Figure 7E). Notably, supplementation with Mg^2+^ not only rescued tissue necrosis caused by LPS but also resulted in the formation of polarized cells near the dentin, revealing a connection between cells and dentin at the microscopic level, which was similar to the arrangement of odontoblasts in normal dental pulp tissue (Figure 7E). DSPP staining revealed that the expression level of the LPS + Mg^2+^ group was greater compared to the LPS group but lower than the control group. Notably, the degree of microvessel formation (black boxes) in the LPS + Mg^2+^ group (indicated by HE staining) resembled that in the natural pulp tissue (Figure 7E). EMCN staining also revealed the ability to promote angiogenesis. Both in vitro and in vivo experiments demonstrated the cytoprotective effects of Mg^2+^ supplementation. We further analyzed its impact on the expression of Mg^2+^ transporters and revealed that Mg^2+^ supplementation significantly downregulated SLC41A1 while upregulating TRPM7 and MagT1 in cellular and animal models, thus suggesting that Mg^2+^ supplementation can alleviate LPS‐induced failure of regeneration and enable formation of pulp‐like tissues in vivo (Figure S6E–G, Supporting Information).

## 3 Discussion

Despite the paradigm shift achieved by dental pulp regeneration technologies, clinical translation remains hampered by infection‐related regenerative failures. Clinical studies have suggested that most failures are related to incomplete clearance of infections.^[^ ^2^ ^]^ Our study identifies LPS‐induced disruption of Mg^2^⁺ homeostasis via SLC41A1 upregulation, along with its downstream effects of mitochondrial permeability transition and pyroptosis in dental stem cells, as a previously unrecognized mechanism in pulp regeneration failure. Animal studies have revealed that LPS‐induced failure of dental pulp regeneration is correlated with mitochondrial damage, and in vitro studies have revealed that disruption of aerobic respiration critically impairs the differentiation capacity of DPSCs. Based on these findings, we hypothesized that damaged mitochondria are unable to provide sufficient energy for cells to regenerate pulp tissue and that the release of mtDAMPs could also affect the fate of stem cells. Further investigation revealed that LPS stimulation of dental stem cells upregulated the Mg^2+^ efflux transporter SLC41A1 on the cell membrane. After SLC41A1 was overexpressed, [Mg^2+^]_i_ decreased, and mtDAMPs were released. The ROS subsequently attacked the mitochondria, leading to MMP collapse. Afterward, mtDNA induced upregulation of the AIM2 inflammasome, thereby activating GSDMD‐mediated pyroptosis, causing RCD in dental stem cells and resulting in pulp regeneration failure. Mg^2+^ is a key ion that regulates cell metabolism, and decreases in [Mg^2+^]_i_ may affect cell energy metabolism.^[^ ^28^ ^]^ Therefore, Mg^2+^ may be a key bridge in causing mitochondrial damage and cell death. Via inhibitor screening, the mPTP on the inner membrane of mitochondria was determined as the key mediator of mtDAMPs release, and Co‐IP confirmed that the binding ability between CypD and OSCP was enhanced under low [Mg^2+^]_i_ conditions, consequently inducing the opening of the mPTP. Interestingly, exogenous Mg^2+^ supplementation alleviated the binding between CypD and OSCP, and the mPTP subsequently closed. Then, mitochondrial damage was alleviated, and pyroptosis mediated by the mtDNA/AIM2/GSDMD pathway was inhibited. In vivo experiments have also demonstrated that exogenous Mg^2+^ can counteract LPS‐induced pulp regeneration failure.

The solute carrier (SLC) superfamily, encompassing > 400 transmembrane transporters, orchestrates cellular homeostasis via substrate‐specific transport of metal ions, metabolites, and xenobiotics.^[^ ^29^ ^]^ For example, SLC7A11 is a specific amino acid transporter and a key regulator of ferroptosis. Downregulation of SLC7A11 can lead to a decrease in intracellular cysteine levels and depletion of glutathione biosynthesis by inhibiting the cysteine metabolism pathway, indirectly inhibiting the activity of glutathione peroxidase 4 and causing lipid peroxidation accumulation, thereby ultimately inducing ferroptosis.^[^ ^30^ ^]^ Iron, as an essential element in humans, critically contributes to ferroptosis when its dyshomeostasis leads to ROS accumulation via the Fenton reaction.^[^ ^31^ ^]^ Unlike SLC7A11 in ferroptosis, SLC41A1 links Mg^2^⁺ efflux to mitochondrial dysfunction, highlighting transporter‐specific roles in RCD. External stimuli often lead to changes in ion homeostasis, whereas regulatory mechanisms of Mg^2+^ homeostasis have received less attention.^[^ ^32^, ^33^ ^]^ This discovery revealed how inflammatory stimuli hijack ion transport systems to disrupt mitochondria. Physiologically, all ATPase reactions require Mg^2+^, which can regulate glucose, lipid, and protein metabolism.^[^ ^34^, ^35^ ^]^ Hypomagnesemia due to impaired kidney function or insufficient dietary intake often demonstrates lethargy, muscle cramps, or muscle weakness, which may be related to energy metabolism.^[^ ^34^ ^]^ Notably, Mg^2+^ addition to cells improved the decreasing trend in the MMP caused by LPS. This is consistent with a previous study showing that exogenous Mg^2+^ supplementation can also increase ATP levels in rat dorsal root ganglia neurons, confirming the role of Mg^2+^ in improving energy supply.^[^ ^36^ ^]^ Ca^2+^ is an important signaling molecule within cells, and calcium oscillations exhibit rapid and intense behavior, which is sufficient to cause significant cellular activity changes.^[^ ^37^ ^]^ However, this contrasts with the pattern of [Mg^2+^]_i_ modulation, wherein fluctuations in [Mg^2+^]_i_ exhibit slow and persistent characteristics.

Afterward, we inferred that Mg^2+^ could affect the opening and closing of the mPTP. Mg^2+^ is believed to exhibit antagonistic effects on Ca^2+^, and the regulation of mPTP opening and closing by Mg^2+^ is believed to result from competitive inhibition with Ca^2+^.^[^ ^38^ ^]^ However, previous research suggests that Mg^2+^ may independently affect the function of mPTP. When cells exhibit low [Mg^2+^]_i_, the sensitivity of the mPTP can increase, resulting in reversible opening.^[^ ^26^ ^]^ The key to the formation of the mPTP is F1F0‐ATP synthase, which is mainly responsible for ATP synthesis. Mg^2+^ is the key cofactor for ATP synthesis, and OSCP is the key protein composed of F1F0‐ATP synthase. OSCP is also considered a potential target for Mg^2+^.^[^ ^39^, ^40^ ^]^ Molecular docking revealed that the binding energy between CypD and OSCP was greater than that between CypD and OSCP‐Mg^2+^. This result suggests that when cellular Mg^2+^ homeostasis is disrupted and [Mg^2+^]_i_ decreases, CypD is more likely to bind to OSCP, thus leading to opening of the mPTP. This opening was reversible and validated in subsequent rescue experiments. The supplementation of Mg^2+^ also has an impact on the transporters’ gene. The decreased SLC41A1 expression after Mg^2+^ supplementation may be attributed to activating STAT3 transcriptional activity, which disrupts the STAT3/STAT5 transcriptional equilibrium, thereby leading to SLC41A1 downregulation.^[^ ^41^, ^42^ ^]^ Zhang et al. suggested that increased extracellular Mg^2+^ induces MagT1 and TRPM7 to mediate Mg^2+^ cellular entry.^[^ ^36^ ^]^ Both in vitro and in vivo experiments demonstrated concurrent upregulation of MagT1 and TRPM7, which is consistent with previous reports.^[^ ^43^, ^44^ ^]^

Notably, the activity of mitochondria was increased in the early stage of the directed differentiation of dental stem cells. Mitochondria in undifferentiated mesenchymal cells largely localize around the nucleus.^[^ ^45^ ^]^ Upon initiation of the differentiation process, mitochondria in mesenchymal stem cells are activated via a yet unknown mechanism, and oxidative phosphorylation becomes the major ATP source.^[^ ^46^ ^]^ Although their quantity change during differentiation, mitochondria play a role in regulating the fate of stem cells, as reflected by their ability to provide energy support and produce metabolites for directed differentiation.^[^ ^47^ ^]^ For example, decreased H_2_O_2_ levels in mitochondria cause increased quiescence and defects in stem cell differentiation, which could be rescued by pharmacologically increasing mitochondrial H_2_O_2_ levels.^[^ ^48^ ^]^ The regulation of mitochondrial homeostasis has been regarded as a new target for stem cell therapy. In our research, we restored Mg^2+^ homeostasis to regulate mitochondrial functional defects; when mitochondrial damage was reversed, stem cells demonstrated greater differentiation potential.

## 4 Conclusion

The present study links Mg^2+^ homeostasis, mitochondria, and pyroptosis, thus providing a new perspective on the pathological mechanism of dental pulp regeneration failure. These findings provide a further understanding of the opening and closing mechanisms of the mPTP, which may contribute to the development of targeted treatment strategies for mPTP‐related diseases.

## 5 Experimental Section

### Extraction and Cultivation of Primary Cells

Human dental pulp and apical papilla were aseptically isolated from healthy premolars or molars obtained under Institutional Review Boards of the Affiliated Stomatology Hospital of Tongji University approved protocols (No. 2022‐DW‐81) with donor informed consent. The tissue was separated and digested in PBS containing type I collagenase (3 mg mL^−1^, Sigma–Aldrich, C0130) for 25 min at 37 °C. Cells were expanded in α‐MEM basal medium (HyClone, SH30265.01B) supplemented with 10% fetal bovine serum (Every Green, 13011–8611) and 1% penicillin‐streptomycin (Gibco, 15 140 122), with medium refreshed every 3 days. Cells at passages 2–7 (P2‐P7) were used for subsequent experiments. For the osteogenic and dentinogenic induction, cells were cultured in the medium containing sodium β‐glycerophosphate (10 mM, Sigma–Aldrich,), ascorbic acid (50 µg mL^−1^, Sigma–Aldrich), and dexamethasone (Sigma–Aldrich, 100 nM) for indicated days. For angiogenic induction, the α‐MEM medium was supplemented with the following components: 50 µg mL^−1^ VEGF (Novoprotein, C744), 20 µg mL^−1^ bFGF (Novoprotein, C046), 100 nM dexamethasone, 5% FBS, and 1% penicillin‐streptomycin. The chondrogenic differentiation medium consisted of high‐glucose DMEM (HyClone, SH30081.01), supplemented with 20 ng mL^−1^ TGF‐β3 (Abbkine, PRP1046), 100 nM dexamethasone, 1% ITS (Beyotime, C0341), 50 µg mL^−1^ ascorbic acid, 1 mM sodium pyruvate (Beyotime, ST1663), and 40 µg mL^−1^ proline (Beyotime, ST1500). Following a 2‐week induction period, the chondrogenic pellets were fixed, cryosectioned, and stained with Alcian blue (Solarbio, G1560). Adipogenic differentiation was induced and tested using the Human Stem Cell Adipogenic Differentiation Kit (OriCell, HUXXC‐90031).

### Cell Viability Analysis

DPSCs or SCAP were seeded in 96‐well plates at 2 × 10^3^ cells per well. After incubation, 10 µL CCK‐8 reagent (Yeasen, 40203ES60) was added per well and incubated for 1 h at 37 °C. Absorbance at 450 nm was measured using a microplate reader (BioTek Synergy H1).

### Real‐Time PCR

Total RNA was isolated from DPSCs or SCAP using RNAiso Plus (Takara, 9109), followed by cDNA synthesis with PrimeScript RT Master Mix (Takara, RR036A). RT‐qPCR was conducted using Hieff qPCR SYBR Green Master Mix (Yeasen, 11201ES03) and was performed on QuantStudio 5 (Applied Biosystems). Relative gene expression was normalized to *ACTB* as an internal control and calculated using the 2^‐ΔΔCt method. All experiments were performed at least in triplicate with technical replicates. The primer sequences are listed in Table S1 (Supporting Information).

### Establishment of Rat Model

Incisor injury model. Surgical procedures were performed on four‐week‐old Sprague‐Dawley rats under isoflurane anesthesia. The left mandibular incisor was surgically sectioned at the gingival level. Then we used the K file to destroy the pulp tissue, from 10 # to 30 #, with an average working length of 20.7 mm. Experimental compounds (CCCP 500 µм, Yeasen, 40333ES60; 2‐DG 20 mM, MCE, HY‐13966; LPS 011:B4 10 mg mL^−1^, Sigma–Aldrich, L4391) were directly injected into pulp cavity (each group *n* = 5). The cut‐edge is sealed with dental resin (3 m, Z350 XT).

Magnesium‐deficient model. Three‐week‐old Sprague‐Dawley rats were fed magnesium‐free diet for four weeks (*n* = 5). Magnesium‐free feed was customized from Jiangsu XieTong Pharmaceutical Bioengineering Co., Ltd. The persistent dermal ulcerations with delayed healing indicated successful modeling. Age‐matched controls (*n* = 5) received a standard diet. This experiment has been approved by the Institutional Review Boards of the Affiliated Stomatology Hospital of Tongji University (No. 2022‐DW‐88).

### Isolation of Mitochondria and Measurement of mtDNA

Briefly, to isolate the mitochondrial fraction and nonmitochondrial fraction, cells were washed and resuspended in ice‐cold hypotonic buffer supplemented with protease inhibitors and were then lysed by Dounce grinder. Cell lysates were centrifuged at 500 g for 10 min at 4 °C to remove nuclear pellets, and the supernatants were then centrifuged at 5000 g for 10 min at 4 °C to obtain the mitochondrial fraction and nonmitochondrial fraction. mtDNA was extracted from these fractions and subjected to qPCR analysis with primers corresponding to the D‐loop in mtDNA. The ratio of the nonmitochondrial fraction and mitochondrial fraction in each sample was used to quantitate the relative release of mtDNA from mitochondria into the cytosol.^[^ ^49^ ^]^

D‐loop Forward: 5‘‐TTATCGCACCTACGTTCAATATTACAG‐3′

D‐loop Reverse: 5‘‐TCTGTGTGGAAAGTGGCTGTG‐3′

### Transmission Electron Microscopy

The dental pulp samples from the apical bud of rat incisor were fixed in 2.5% glutaraldehyde (Sigma–Aldrich, USA) for 1 h followed by 2% osmium tetraoxide for 2 h. After washing with water, the cell samples were stained with 0.5% uranyl acetate for 12 h, followed by dehydration and polymerization. Ultrathin sections were cut at thicknesses of 70–90 nm with an ultramicrotome (Leica, EM UC7) and imaged with a Tecnai G2 TWIN transmission electron microscope.

### Bioinformatics Analysis of GEO Database and Transcriptome Sequencing Analysis

The microarray datasets GSE174260 was downloaded from the GEO database (https://www.ncbi.nlm.nih.gov/geo/). For processing the microarray datasets, match the probe and gene names based on the provided annotation information for each GPL platform. Normalize the expression matrix using the normalizeBetweenArrays function in the R/Bioconductor limma package (v3.50.0) and perform log2 transformation on datasets requiring it. Utilizing the GSEA algorithm enabled the computation of enrichment scores that reflect the overall activity level of the gene set.

For RNA‐seq, after 24 h cell culture, total RNA of P2 DPSCs of control, LPS (5 µg mL^−1^, L6529, Sigma–Aldrich) and LPS (5 µg mL^−1^) + MgCl_2_ (5 mM, 442 615, Sigma–Aldrich) groups were collected and measured using the NanoDrop One Microvolume UV–vis Spectrophotometer (Thermo Scientific). By using Illumina HiSeq X10 (Illumina, San Diego, CA), the following RNA reverse transcription, library construction and the sequencing were accomplished at Majorbio Bio‐pharm Biotechnology Co., Ltd (Shanghai, China). Under the orientation mode, above separated clean reads were subjected to the sequencing alignment with the reference genome of Homo_sapiens v.GRCh38 (http://asia.ensembl.org/Homo_sapiens/Info/Index) by using TopHat (http://tophat.cbcb.umd.edu/, version2.1.1) software. The bioinformatic data were analyzed on the Majorbio Cloud Platform (Majorbio Bio‐pharm Biotechnology, China).

### Plasmid Construction and Transfection

The shRNA of *TRPM7* and *SLC41A1* were designed by GeneChem Corporation and ligated to plasmids GV112. The sequences are as follows. Cell transfection used Lipofectamine 3000 reagent (Thermo Scientific, L3000008).

The sh*TRPM7*: Forward oligo: 5‘ CCGG—CCACCAAAGAATCAGAATCAA—CTCGAG—TTGATTCTGATTCTTTGGTGG—TTTTTG 3′ Reverse oligo: 5‘ AATTCAAAAA—CCACCAAAGAATCAGAATCAA—CTCGAG—TTGATTCTGATTCTTTGGTGG 3′.

The sh*SLC41A1*: Forward oligo: 5‘ CCGG—TGCAGCCAACATTGGACACAT—CTCGAG—ATGTGTCCAATGTTGGCTGCA—TTTTTG 3′ Reverse oligo: 5‘ AATTCAAAAA—TGCAGCCAACATTGGACACAT—CTCGAG—ATGTGTCCAATGTTGGCTGCA 3′.

### Inductively Coupled Plasma‐Mass Spectrometry of Mg^2+^ in Dental Stem Cells

DPSCs and SCAP were utilized. Cells were transfected and harvested 24 h post‐transfection. The cells were treated with 5 mM Mg^2^⁺ immediately after transfection. All samples were adjusted to 1 × 10^7^ cells per group, snap‐frozen in liquid nitrogen, and subjected to elemental analysis via inductively coupled plasma‐mass spectrometry (PANOMIX Biomedical Tech Co., Ltd., Suzhou, China). The measurements were carried out with Thermo Fisher Scientific iCAP TQ ICP‐MS/MS.

### Immunohistochemistry and Immunofluorescence

Rats were euthanized via isoflurane overdose and transcardially perfused with ice‐cold PBS followed by 4% paraformaldehyde (PFA). Mandibles were dissected, post‐fixed in 4% PFA at 4 °C for overnight, and decalcified in 10% EDTA (pH 7.4) for 8 weeks with weekly solution replacement. Decalcified tissues were paraffin‐embedded, and sagittal sections (4 µm thickness) were prepared. For immunohistochemistry, 3% hydrogen peroxide was performed for 15 min to inhibit endogenous peroxidase activity. Sections were incubated with goat serum (Maxim Biotechnology, SP KIT‐B2) for 1 h to block non‐specific binding site, and then the following anti bodies: anti‐DSPP (1:200, absin, abs118471), anti‐AIM2 (1:200, proteintech, 20590‐1‐AP), anti‐GSDMD‐N (1:200, abcam, ab215203), anti‐SLC41A1 (1:200, NOVUS, NBP1‐82652) were incubated overnight at 4 °C. The sections were then incubated with appropriate secondary antibodies and visualized with a DAB detection kit (DAB‐0031, Maxim Biotechnology). Negative controls were conducted by omitting the primary antibody. Hematoxylin counterstaining was performed (BBI CO., LTD). For tissue immunofluorescent staining, antigen retrieval was performed with citrate; sections were blocked in goat serum for 1 h and incubated with anti‐CD90 (1:200, Affinity, DF6849), anti‐Endomucin (1:200, Santa Cruz, sc‐65495) overnight at 4 °C. And appropriate secondary anti bodies conjugated with YSFluor 488 Doneky Anti‐Rabbit lgG (H + L) (1:400, Yeasen, 34206ES60) and YSFluor 594 Doneky Anti‐Rabbit lgG(H + L) (1:400, Yeasen, 34212ES60) were used, respectively. Sections were subsequently stained wi th DAPI (1:2000, Yeasen, 40728ES03).

EdU staining was performed using the BeyClick EdU‐594 cell proliferation assay kit (Beyotime, C0078L). 2 h before euthanasia of experimental animals, EdU (50 mg kg^−1^) was injected intraperitoneally. The sample processing is as described earlier, and the EdU reaction reagent is prepared according to the protocol.

In vivo ROS test. In the 3‐day rat experimental group model, the ROS indicator DCFH‐DA (Beyotime, S0033) was injected through the tail vein, and the contralateral incisor served as a self‐control. Following euthanasia of the rats, dental pulp samples were collected and cryosectioned at a thickness of 8 µm.

### Cell Staining

ALP staining was performed using the BCIP/NBT Alkaline Phosphatase Color Development Kit (Beyotime, C3206). After DPSCs and SCAP underwent osteogenic differentiation induction, the cells were collected and fixed with 4% PFA. Then the cells were stained with the ALP working buffer. Masson staining was performed using a Masson's Trichrome Stain Kit (Solarbio, G1340).

Mitochondrial morphology visualization was performed using MitoTracker Red CMXRos (Beyotime, C1035) staining. Following cell fixation with 4% PFA, specimens were incubated with 100 nM MitoTracker working solution for 30 min at 37 °C. Subsequent PBS washes (3 × 3 min) were conducted prior to nuclear counterstaining with DAPI (Yeasen, 40728ES03) at 1:2000 dilution for 5 min, followed by PBS rinses. High‐resolution z‐stack imaging was acquired using a Nikon A1+ confocal laser scanning microscope equipped with a 60 × oil‐immersion objective (NA 1.4), employing 0.5 µm optical sectioning intervals. 3D mitochondrial reconstruction and morphometric analysis were executed through Fiji ImageJ software utilizing the Mitochondria Analyzer plugin with default parameters.

Magnesium ion Staining. Cells were incubated with 5 µм Mag‐Fluo‐4 AM (MKBio, MX4544) for 30 min, washed thrice with PBS, the fluorescence lifetime imaging microscopy (FLIM) was captured via Leica Stellaris 8.

Calcium ion Staining. Cells were incubated with 5 µм Fluo‐4 AM (Yeasen, 40704ES50) at 37 °C for 30 min, washed thrice with PBS, and calcium signals were captured via fluorescence microscopy.

### Western Blot

Total protein was isolated using RIPA (Beyotime, P0013B) buffer containing protease inhibitor cocktail (Beyotime, P1008). The supernatant of the cell lysate was collected through centrifugation at 12000 rpm for 10 min at 4 °C. Then the loading buffer (Beyotime, P0015) was added to the protein solution and denatured at 100 °C for 5 min. Proteins were separated by SDS‐PAGE gel electrophoresis and then transferred onto a Nitrocellulose membrane (HATF00010, Millipore). After blocking the membranes with 5% bovine serum albumin solution for 1 h, the primary antibodies were incubated overnight at 4 °C followed by secondary antibodies for 1 h at room temperature. The blots were visualized using Amersham ImageQuant 800 (Cytiva). The primary antibodies included anti‐β‐actin (1:1000, abcam, mAbcam 8224), anti‐TRPM7 (1:1000, proteintech, 55251‐1‐AP), anti‐SLC41A1 (1:1000, NBP1‐82652, NOVUS), anti‐AIM2 (1:1000, proteintech, 20590‐1‐AP), anti‐GSDMD (1:1000, proteintech, 20770‐1‐AP).

### Flow Cytometry Analysis

Stem cell identification: After cell fixation with 4% PFA, flow cytometry antibody staining was performed, including FITC anti‐human CD44 Antibody (Biolegend, 397 517), FITC anti‐human CD90 Antibody (Biolegend, 328 107), APC anti‐human CD105 Antibody (Biolegend, 323 207), FITC anti‐human CD34 Antibody (Biolegend, 343 503), FITC anti‐human CD45 (Biolegend, 982 316).

Fluorescent indicator staining: Prior to staining, cells were digested with trypsin and 0.5 mL of dye was added at a concentration of 1 × 10^5^ cells according to the protocol instructions. mPTP assay kit (Biosharp, BL928A), JC‐1 kit (Yeasen, 40706ES60), DCFH‐DA dye (Beyotime, S0033S), and Mag‐Fluo‐4 AM (MKBio, MX4544) were used for staining analysis. Subsequently, flow cytometry analysis was performed. The pharmacological inhibitors used in this study were: Cyclosporin A (2 µм, MedChemExpress, HY‐B0579), BA1 (500 nM, TargetMol, T5210), Quinidine (100 µм, MedChemExpress, HY‐B1751), N‐acetylcysteine (5 mM, Beyotime, S0077).

### Dual‐Luciferase Reporter Assay

Human *SLC41A1* promoter with wild‐type or mutant binding site was amplified from genomic DNA by PCR primers, subcloned into luciferase reporter vector pGL4.15 [luc2P/Hygro] Vecto (Promega), and confirmed by sequencing. Cells were co‐transfected with these established luciferase reporter vectors (30 ng) and Renilla luciferase reporter vector pRL‐SV40 (10 ng; Promega) as an internal reference. Dual‐luciferase assay was performed using a luminometer (Berthold Tech, Lumat LB9507). the *STAT5A* plasmids were purchased from Tsingke Biotechnology Co.,Ltd (Beijing, China).

### Molecular Docking

The molecular structures of OSCP (q8wvf1) and CypD (3qyu) were obtained from the Uniprotein database (https://www.uniprot.org/), and performed molecular docking using Alphafold 3 software with default parameters. Alphafold 3 calculates the optimal binding between Mg^2+^ and OSCP. After docking, the complex was obtained, and PDBePISA was used to evaluate the protein interaction binding energy and interaction (https://www.ebi.ac.uk/msd‐srv/prot_int/pistart.html).

### Co‐Immunoprecipitation

To perform direct co‐immunoprecipitation of intracellular proteins, cells were cultured in 10 cm dishes until they reached 80%–90% confluency. The cells were then lysed using RIPA lysis buffer (Beyotime, P0013B) on ice for 20 min. The lysates were centrifuged at 12000 g for 10 min at 4 °C and the supernatant was collected. The total protein content in the supernatant was quantified using a BCA kit. Approximately 2000 µg of proteins were transferred to a fresh 1.5 mL tube and cell lysates were coimmunoprecipitated with an anti‐CypD (Proteintech, 18466‐1‐AP) antibody or isotype antibody with protein A/G beads (Beyotime, P2108) at 4 °C overnight on a rotator. The beads were washed by 100 µL 1 × loading buffer for 5 min at 95 °C and then perform immunoblotting detection with anti‐CypD and anti‐OSCP (1:1000, sc‐365162, Santa Cruz).

### TDM Subcutaneous Transplantation in Nude Mice

Clinically extracted human premolars or molars were collected. Periodontal ligament tissues were carefully scraped away along with partial removal of outer cementum, inner dental pulp tissue, and predentin. High‐speed air turbine handpiece was used to cut the root canal into pieces of 4 mm in length. Next, the root canal pieces were concussed for three times (5 min each time) by an ultrasonic cleaner before further exposed to a series of decreasing EDTA solutions (from 17% to 5%) for 5 min and washed with deionized water for 5 min (three times). Last, the prepared TDM was in culture medium with penicillin (50 U mL^−1^) and streptomycin (50 mg mL^−1^) at 4 °C. Approximately 1 × 10^7^ DPSCs were resuspended in 1 mL Matrigel (Corning, 354 262) and injected into the dental segment. The TDM end was sealed with iRoot BP plus (IBC, IRBPP 4610 U5), and was subcutaneously implanted in 4‐week‐old BALB/c‐nu mice for 4 weeks. This experiment has been approved by the Ethics Committee of Tongji University Experimental Animal Center (No. TJBD00324101).

### Statistical Analysis

All the data are presented as the mean ± standard deviation (SD) of at least three independent experiments. Two‐tailed Student's t test and one‐way or two‐way ANOVA were used to assess the statistical significance of the differences. Statistical analysis was performed with GraphPad Prism 8.0 software, and *p* < 0.05 was considered statistically significant.

## Conflict of Interest

The authors declare no conflict of interest.

## Author Contributions

Conceptualization was carried out by B.J., Y.L., and C.L.; Methodology was applied by Y.L., C.S., L.Z., X.H., Y.Y., C.L., and B.Y.; Investigation was conducted by Y.L., L.C., M.S., S.J., A.M., and C.L.; Visualization was carried out by Y.L., C.S., and L.Z.; Funding acquisition was performed by Y.Y., C.L., and B.J.; Project administration was carried out by Y.Y., C.L., and B.J.; Supervision was dealt with by B.J.; Original draft was written by Y.L.; Writing – review & editing was conducted by all authors.

## Supporting information



Supporting Information

## Data Availability

The data that support the findings of this study are available from the corresponding author upon reasonable request.
