# Supplementary material for: LPS‐Induced Mitochondrial Damage via SLC41A1‐Mediated Magnesium Ion Efflux Leads to the Pyroptosis of Dental Stem Cells
Source: Adv Sci (Weinh). 2025 Aug 19;12(42):e05666. doi: 10.1002/advs.202505666 (PMC12622442; doi:10.1002/advs.202505666)

Supporting Information

LPS-induced mitochondrial damage via SLC41A1-mediated magnesium ion efflux leads to the pyroptosis of dental stem cells

*Yuan Liu, Chenyu Song, Liyuan Zhang, Xue Han, Chaoyuan Li, Yanhong Yan, Ludan Xing, Mengting Si, Bo Yang, Lingyuan Cheng, Akimi Muramatsu, Beizhan Jiang**


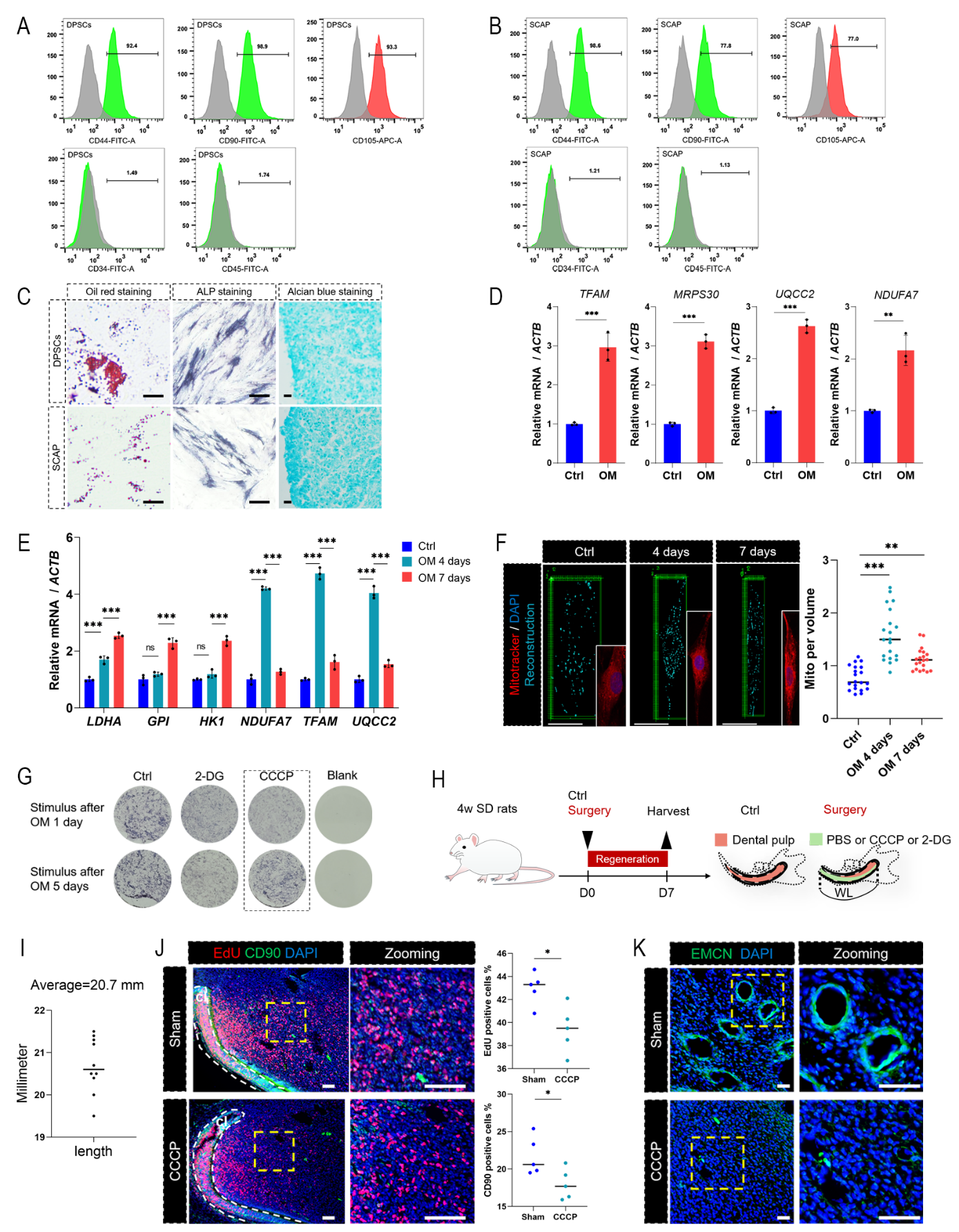


**Figure S1.** Identification of dental stem cells and analysis of SCAP-related respiratory metabolism gene expression. A) Flow cytometry identification of DPSCs using stem cell markers CD44 (+), CD90 (+), CD105 (+), CD34 (-), CD45 (-). B) Flow cytometry identification of SCAP, stem cell markers CD44 (+), CD90 (+), CD105 (+), CD34 (-), CD45 (-). C) Adipogenic, osteogenic, and chondrogenic induction of DPSCs and SCAP demonstrated their multilineage differentiation potential. Bar = 10 μm. D) Following 3 days of osteogenic differentiation induction, SCAP demonstrated significant upregulation of mitochondrial translation-related genes, including *MRPS30*, *UQCC2*, *NDUFA7*, and *TFAM* (each group n = 3). E) The expression of representative genes of glycolysis (*LDHA*, *GPI*, *HK1*) and aerobic respiration (*NDUFA7*, *TFAM*, *UQCC2*) for SCAP (each group n = 3). F) Representative image of MitoTracker staining during osteogenic differentiation of SCAP. The mitochondria increased at day 4 of induction and decreased at day 7. Bar = 10 μm. G) ALP staining to SCAP after osteogenic differentiation (each group n = 5). Interventions were conducted at day 1 or 5 of osteogenic induction, respectively. The CCCP (10 μM) intervention at day 1 of induction significantly inhibited osteogenic differentiation. The 2-DG (2 mM) intervention has no significant difference at day 1 or day 5. H) Schematic diagram depicting the construction of rat incisor pulp injury model, with injection of PBS or inhibitors into the root canal. Sample were collected samples on day 7 after surgery. I) Analysis of working length in 4-week rats from gingival margin to cervical loop. J) Representative immunofluorescence analysis of rat incisor pulp stem cell niches (each group n = 5). EdU labeled proliferating cells and CD90 labeled stem cells. The proliferated cells and the expression of CD90 were reduced in the CCCP group. Statistical analysis of EdU⁺ and CD90⁺ cells on the right. Scale bar = 100 μm. cl: cervical loop. K) Representative immunofluorescence analysis was performed on rat incisor pulp, and staining analysis was performed using the vascular marker EMCN. The expression of CCCP group was weaker than that of the sham group (each group n = 5). Scale bar = 100 μm. ns, no significance; **p*<0.05, ***p*<0.01, ****p*<0.001.


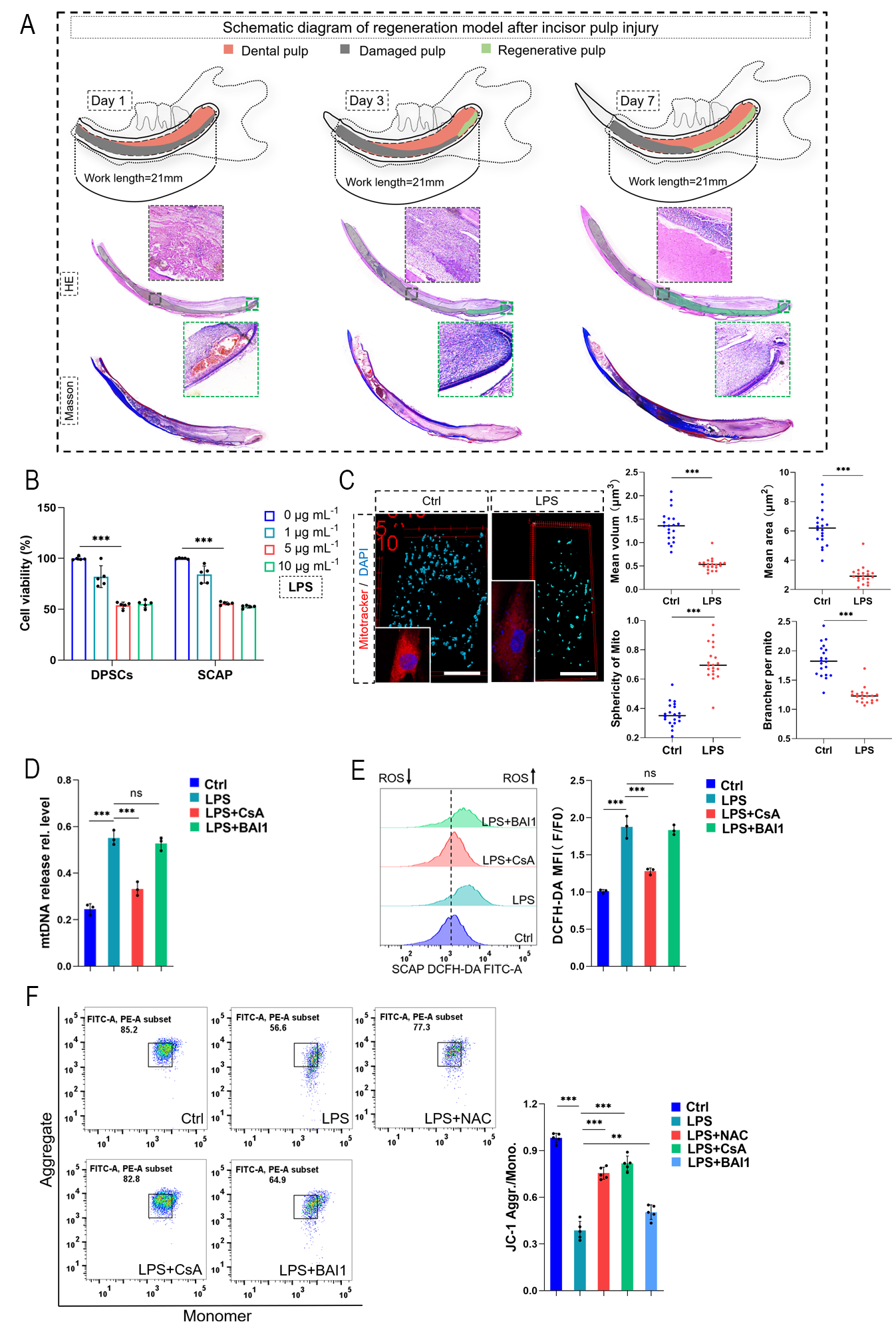


**Figure S2.** LPS stimulation caused mitochondrial damage in SCAP. A) Schematic diagram of regeneration model after incisor pulp injury. H&E and Masson staining revealed postoperative changes at days 1, 3, and 7. Due to the presence of apical stem cell niches, regenerated pulp tissue progressively replaced damaged tissue, ultimately leading to restoration of pulp and dentin. B) The cell viability of DPSCs and SCAP was analyzed after 48 hours of stimulation with different concentrations of LPS. The results showed that 5 μg mL^-1^ had a significant impact on the cells (each group n = 5). C) In vitro, SCAP mitochondria were labelled by MitoTracker and performed with mitochondrial morphological analysis. After LPS stimulation, the sphericity increased, indicating mitochondrial expansion from rod-shaped to spherical, while the average volume, average area, and brancher per mitochondria decreased, suggesting mitochondrial fragmentation (each group n = 20). Bar = 10 μm. D) Analysis of cytoplasmic mtDNA release in SCAP. LPS stimulation induced mtDNA release, CsA inhibited its release, but BAI1 had a weaker inhibitory effect (each group n = 3). E) Analysis of SCAP ROS showed that LPS induced an increase in cellular ROS, while CsA could inhibit the increase in ROS. But BAI1 had a weaker inhibitory effect (each group n = 3). F) Representative flow cytometry plots of JC-1 in SCAP. The ratio of aggregate/monomer represented the level of MMP. LPS stimulation caused a decrease in MMP, while CsA inhibited the decrease, but BAI1 had a weaker inhibitory effect. Quantification shown in right (each group n = 5). ns, no significance, ***p*<0.01, ****p*<0.001.


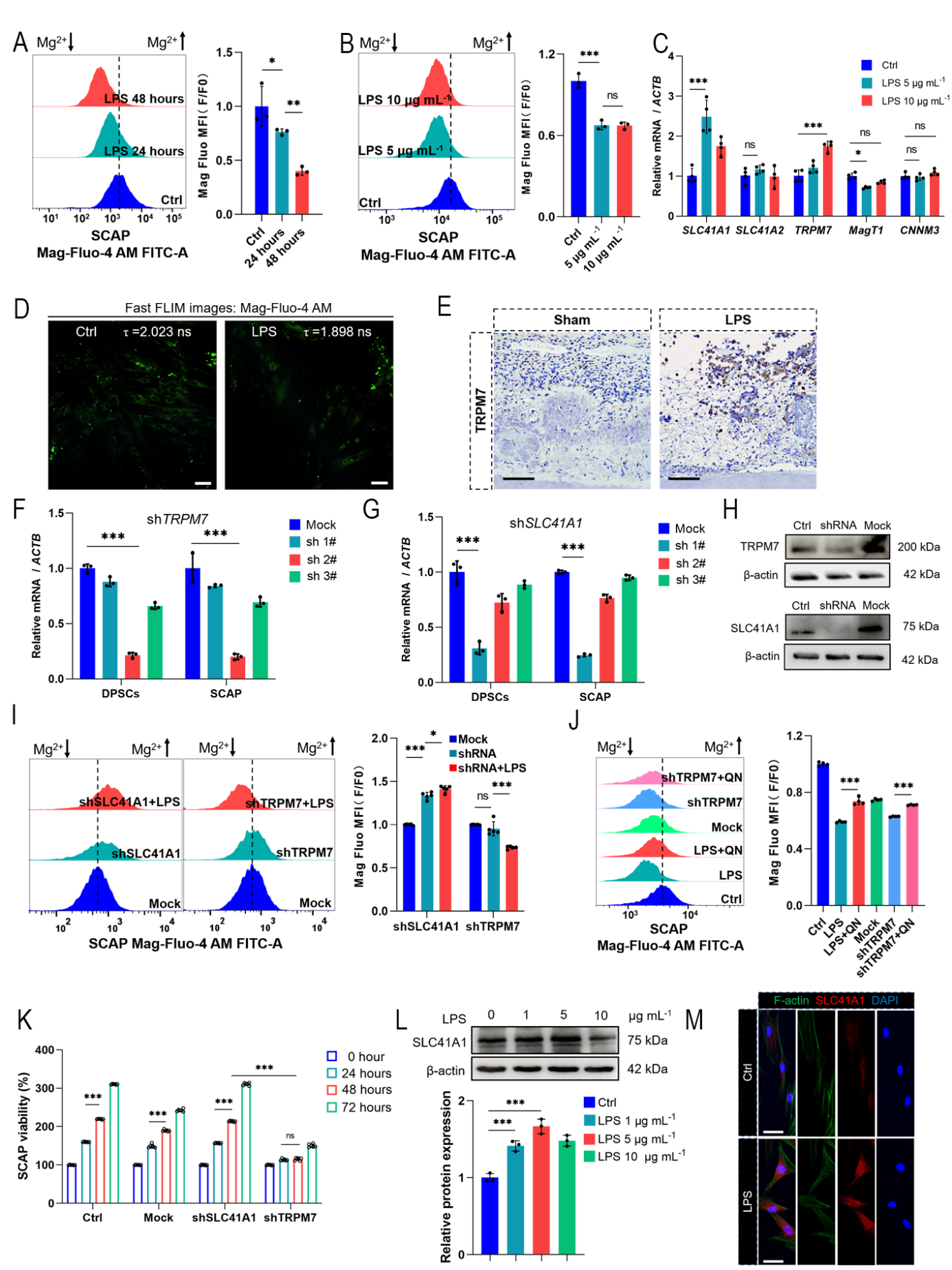


**Figure S3.** The effect of LPS stimulation on the Mg^2+^ homeostasis in SCAP. A) Representative flow cytometry histograms of [Mg^2+^]_i_ (left) and quantification (right). After LPS stimulation, the changes in [Mg^2+^]_i_ were analyzed at 24 and 48 hours, and the [Mg^2+^]_i_ gradually decreased (each group n = 3). B) Representative flow cytometry histograms of [Mg^2+^]_i_ (left) and quantification (right). There was no difference with stimulation of LPS at 5 μg mL^-1^ and 10 μg mL^-1^ (each group n = 3). C) RT-qPCR analysis of gene expression in Mg^2+^ channel expression. *SLC41A1* and *TRPM7* were significantly upregulated after LPS stimulation (each group n = 4). D) immunohistochemistry showing upregulation of TRPM7 in LPS-stimulated group. E) FLIM analysis of Mg²⁺ dynamics in SCAP. F) RT-qPCR validation of sh*TRPM7* knockdown efficiency (each group n = 3). G) RT-qPCR validation of sh*SLC41A1* knockdown efficiency (each group n = 3). H) Immunoblotting validated the efficiency of TRPM7 and SLC41A1 knockdown. I) Representative flow cytometry histograms of [Mg^2+^]_i_ (left) and quantification (right). Analysis of the effect of knocking down TRPM7 and SLC41A1 on cytoplasmic [Mg^2+^]_i_. Knocking down TRPM7 caused a decrease in [Mg^2+^]_i_, which further decreases after LPS stimulation. Knocking down SLC41A1 caused an increase in [Mg^2+^]_i_, but LPS stimulation did not lead to a decrease (each group n = 5). J) Representative flow cytometry histograms of [Mg^2+^]_i_ (left) and quantification (right). Analysis of [Mg^2+^]_i_ after inhibition of SLC41A1 by non-specific sodium channel inhibitor quinidine (QN). Both LPS and TRPM7 knockdown caused a decrease in [Mg^2+^]_i_, but quinidine was able to inhibit the decrease in [Mg^2+^]_i_, indicating that quinidine inhibited the sodium/magnesium transporter (each group n = 4). K) The effect of knocking down TRPM7 and SLC41A1 on cell viability. Knocking down TRPM7 resulted in a significant decrease in viability compared to knocking down SLC41A1, especially after 48 hours of cell culture (each group n = 5). L) Immunofluorescence showing LPS-induced upregulation of SLC41A1. M) In vitro validation of the effect of LPS on SLC41A1 expression. LPS at concentrations of 0, 1, and 5 μg mL^-1^ gradually increased the expression of SLC41A1, but LPS at concentrations of 10 μg mL^-1^ did not further upregulate it (each group n = 3). ns, no significance, **p*<0.05, ***p*<0.01, ****p*<0.001.


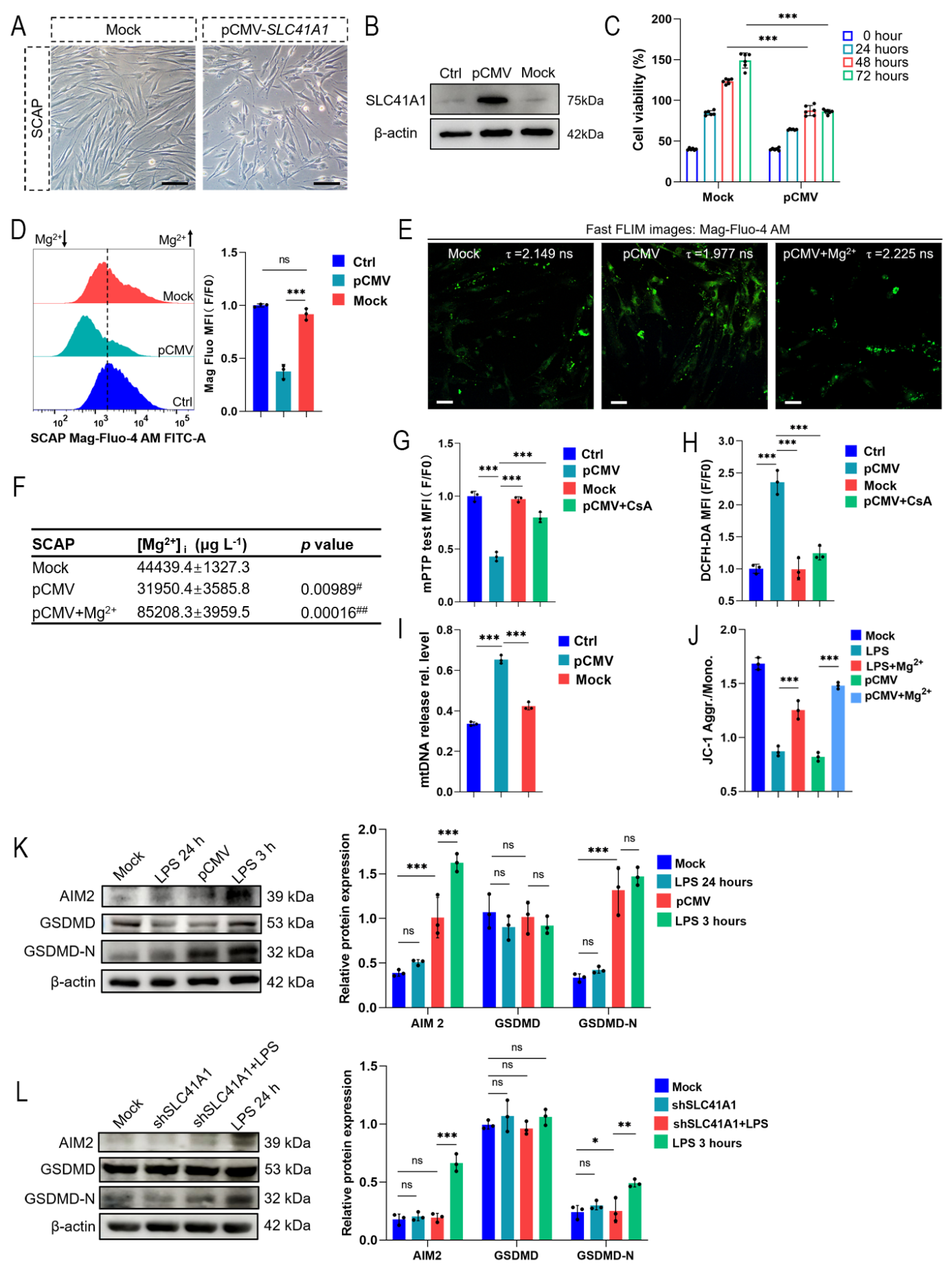


**Figure S4.** Overexpression of SLC41A1 caused mitochondrial damage in SCAP. A) Morphological changes of overexpressing SLC41A1 under bright field. After transfection for 24 hours, the cell expanded and the edges became bright. Bar = 25 μm. B) Immunoblotting validation of SLC41A1 overexpression (pCMV). C) Overexpression of SLC41A1 (pCMV) resulted in a significant decrease in SCAP cell viability after 48 hours (each group n = 6). D) Overexpression of SLC41A1 (pCMV) led to a decrease in intracellular [Mg^2+^]_i_ after 24 hours (each group n = 3). E) FLIM revealed shortened fluorescence lifetime post-transfection (pCMV group) in SCAP, which was prolonged upon Mg²⁺ supplementation. Scale bar = 10 μm. F) ICP-MS detected decreased [Mg^2+^]_i_ levels in SCAP following overexpression, which increased after Mg²⁺ supplementation (each group n = 3; #: pCMV vs. Mock; ##: pCMV + Mg²⁺ vs. Mock). G) Overexpression of SLC41A1 (pCMV) increased the opening of mPTP tested by flow cytometry in the mitochondrial inner membrane of SCAP after 24 hours (each group n = 3). H) Overexpression of SLC41A1 (pCMV) led to an increase in ROS tested by flow cytometry in SCAP after 24 hours (each group n = 3). I) Overexpression of SLC41A1 (pCMV) increased mtDNA release in SCAP after 24 hours (each group n = 3). J) Overexpression of SLC41A1 (pCMV) led to a decrease in MMP tested by flow cytometry in SCAP after 24 hours (each group n = 3). K) Immunoblotting detected the expression of pyroptosis protein in SCAP overexpressing SLC41A1 (pCMV). LPS stimulation after 24 hours and overexpression of SLC41A1 both cause upregulation of AIM2 and GSDMD-N. The LPS-primed 3 hours and ATP-stimulated group was positive control (each group n = 3). L) Immunoblotting validated the expression of pyroptosis after knocking down SLC41A1. After knocking down SLC41A1, the expression of AIM2 and GSDMD-N decreased (each group n = 3). ns, no significance, **p*<0.05, ***p*<0.01, ****p*<0.001.


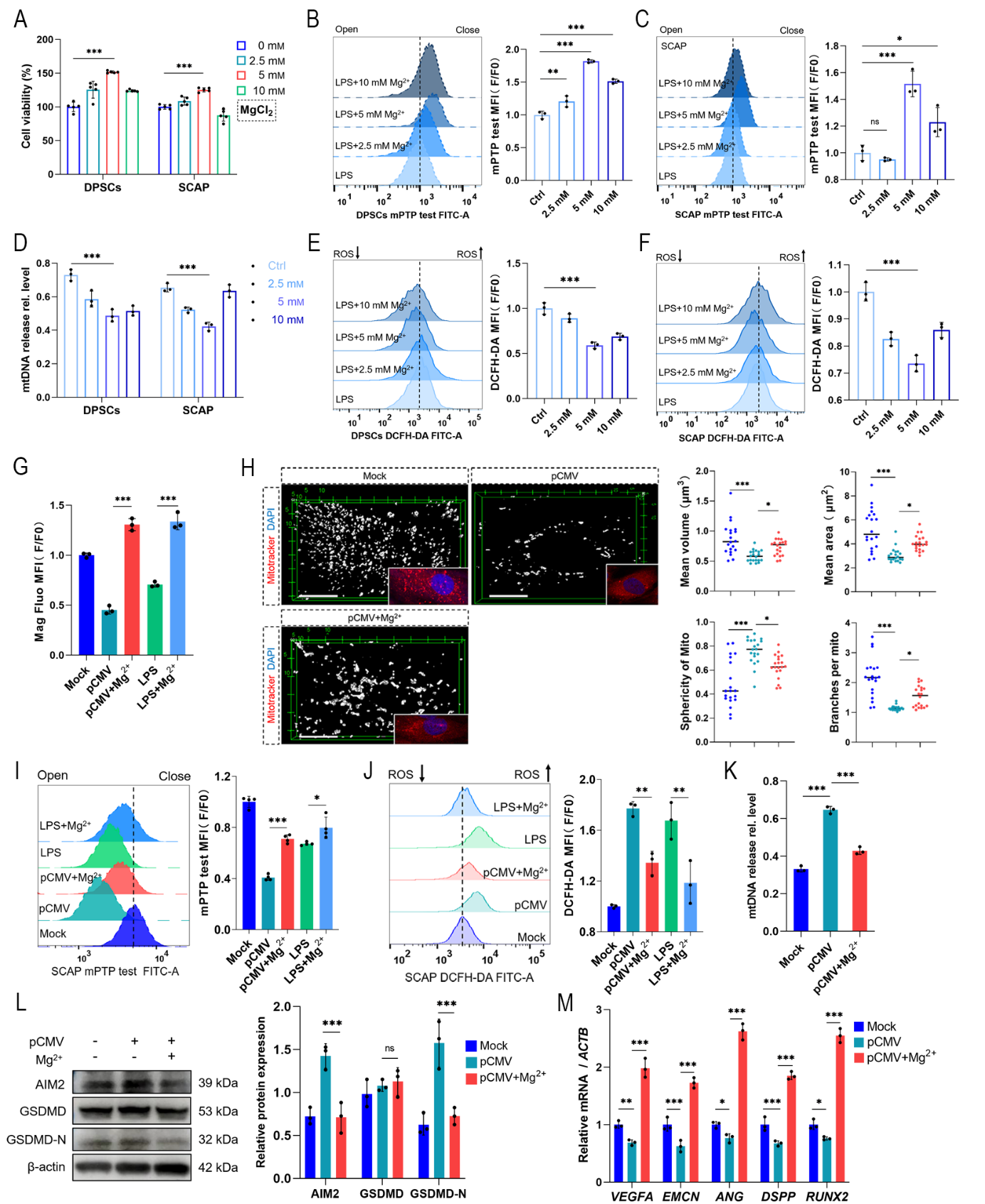


**Figure S5.** Exogenous Mg^2+^ supplement alleviated LPS-induced mitochondrial damage of SCAP. A) Screening of the effect of different concentrations of MgCl_2_ on the viability of dental stem cells (each group n = 5). B) and C) Screening of the effect of different concentrations of Mg^2+^ on mPTP, LPS (5 μg mL^-1^) caused mPTP opening, 5 mM Mg^2+^ significantly reduces opening (each group n = 3). D) Screening of the effect of different concentrations of Mg^2+^ on mtDNA release, LPS (5 μg mL^-1^) caused ROS release, 5 mM Mg^2+^ significantly reduced release (each group n = 3). E) and F) Screening of the effect of different concentrations of Mg^2+^ on ROS release, LPS (5 μg mL^-1^) caused ROS release, 5 mM Mg^2+^ significantly reduced release (each group n = 3). G) Exogenous Mg^2+^ supplement led to an increase in [Mg^2+^]_i_ tested by flow cytometry, resisting the Mg^2+^ efflux caused by the upregulation of SLC41A1 (each group n = 3). H) In vitro, mitochondria were labelled by MitoTracker and performed with mitochondrial morphological analysis. Overexpression of SLC41A1 (pCMV) lead to an increase in sphericity, indicating mitochondrial expansion from rod-shaped to spherical, while the average volume, average area, and brancher per mitochondria decreased, suggesting mitochondrial fragmentation. After exogenous supplement of Mg^2+^, the sphericity decreased, while the average volume, average area, and brancher per mitochondria increased (each group n = 20). Bar = 10 μm. I) Representative flow cytometry histograms of mPTP opening (left) and quantification (right). Exogenous Mg^2+^ supplementation could reduce the mPTP channel open state caused by LPS (24 hours) or overexpression of SLC41A1 (24 hours) (each group n = 4). J) Representative flow cytometry histograms of ROS test (left) and quantification (right). Exogenous Mg^2+^ supplementation could reduce the elevated levels of cytoplasmic ROS caused by LPS (24 hours) or overexpression of SLC41A1 (pCMV, 24 hours) (each group n = 3). K) Exogenous Mg^2+^ supplement could reduce the release of mtDNA caused by LPS (24 hours) or overexpression of SLC41A1 (pCMV, 24 hours) (each group n = 3). L) Exogenous Mg^2+^ supplementation downregulated the upregulation of AIM2 and GSDMD-N caused by overexpression of SLC41A1 (pCMV, 24 hours) (each group n = 3). M) After inducing vascular or dentinogenic differentiation of DPSCs for 7 days, overexpression of SLC41A1 (pCMV, 24 hours) resulted in downregulation of vascular and dentinogenic differentiation genes. Exogenous Mg^2+^ supplement was able to reverse this state (each group n = 3). ns, no significance, **p*<0.05, ***p*<0.01, ****p*<0.001.


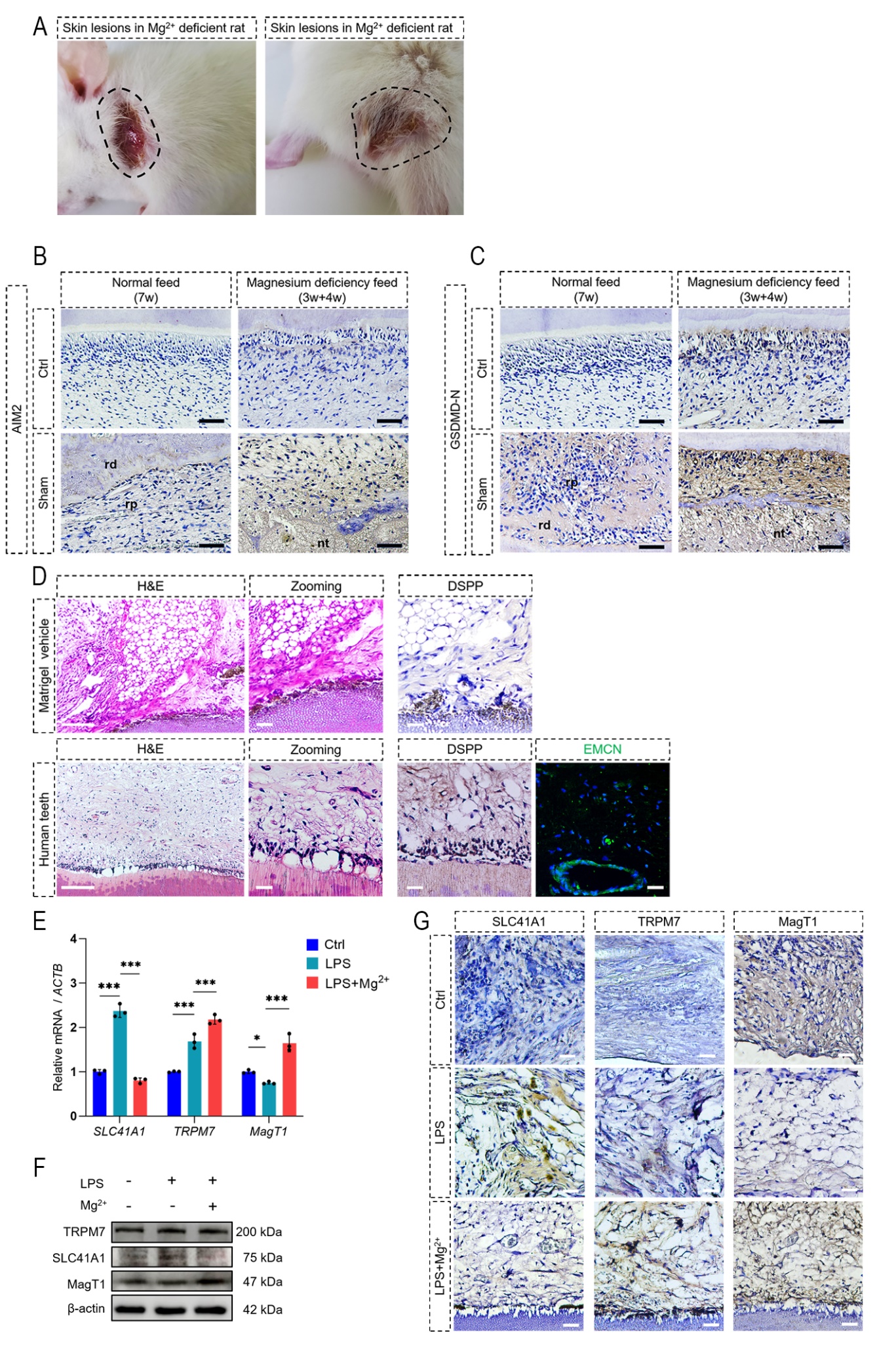


**Figure S6.** In vivo experimental verification of the effect of Mg^2+^ on pulp regeneration. A) Sprague-Dawley rats (3-week-old) fed a magnesium-deficient diet for 4 weeks developed characteristic cutaneous lesions (erythema and scaling) (each group n = 5). B) Compared with the normal control group, magnesium-deficient rats showed AIM2 expression in their dental pulp tissue, especially near the dentin. After damaging the pulp of the incisors, the expression of AIM2 significantly increased in the magnesium-deficient group (each group n = 5). rd: regenerative dentin; rp: regenerative pulp; nt: necrosis tissue. Bar = 100 μm. C) Compared with the normal control group, magnesium-deficient rats showed GSDMD-N expression in their dental pulp tissue, especially near the dentin. After damaging the pulp of the incisors, the expression of GSDMD-N significantly increased in the magnesium-deficient group (each group n = 5). Bar = 100 μm. D) Histological analysis of human teeth (upper). HE staining showed that the dental pulp is a connective tissue containing a large number of dental pulp cells, with odontoblast connections near the dentin and high expression of DSPP. Blood vessels can be seen distributed in the dental pulp tissue. There is a large amount of loose connective tissue in the TDM empty group, with vacuolar changes and no DSPP expression (lower) (each group n = 5). bv: blood vessel. Bar = 100 μm. E) Mg²⁺ supplementation suppresses *SLC41A1* expression while upregulating influx transporters *TRPM7* and *MagT1* by qPCR (each group n = 3). F) Immunoblot analysis of Mg²⁺ transporters following Mg²⁺ supplementation. G) Immunohistochemical analysis of Mg²⁺ transporter expression in TDM models. ns, no significance, **p*<0.05, ***p*<0.01, ****p*<0.001.

Table S1. Primers used for RT-qPCR analysis

| Gene name |  | Primer |
| --- | --- | --- |
| *NDUFA7* | F' | 5'-TCCAAGCGAACTCAGCCTCCTC-3' |
|  | R' | 5'-CCTTCTGCGACGACATGATGATGG-3' |
| *UQCC2* | F' | 5'-GCCAGTGGACGAGACCAAACG-3' |
|  | R' | 5'-TGGGTATTCTCTCCCTCCCGAAAG-3' |
| *TFAM* | F' | 5'-TGGCGTTTCTCCGAAGCATGTG-3' |
|  | R' | 5'-TGCCAAGACAGATGAAAACCACCTC-3' |
| *MRPS30* | F' | 5'-CGGTAGACGAGAAGCTGCGAATC-3' |
|  | R' | 5'-CAGGAACACGGTCTTGGTGAAGTAC-3' |
| *ACTB* | F' | 5'-GGCCAACCGCGAGAAGATGAC-3' |
|  | R' | 5'-GGATAGCACAGCCTGGATAGCAAC-3' |
| *HK1* | F' | 5'-CGTGTCCTTCCTCCTGTCTGAG-3' |
|  | R' | 5'-CTGCTTGCCTCTGTGCGTAAC-3' |
| *GPI* | F' | 5'-GTGATGCCAGAGGTCAACAAGG-3' |
|  | R' | 5'-GGAGCCGCCAATGCCAATG-3' |
| *LDHA* | F' | 5'-GATTCAGCCCGATTCCGTTACC-3' |
|  | R' | 5'-AGAGACACCAGCAACATTCATTCC-3' |
| *SLC41A1* | F' | 5'-CCTGGAACTGAATCACTGGCGATAC-3' |
|  | R' | 5'-ACCTCCCTTGTGGCTGGACTTC-3' |
| *TRPM7* | F' | 5'-ATACCTCCAGCAGCACTCCTCAG-3' |
|  | R' | 5'-CAATCTTTGGTCGGTAGGGCTGTG-3' |
| *SLC41A2* | F' | 5'-GATGGCTGTGGATTGCTGACTG-3' |
|  | R' | 5'-AATGCTGTTAGGTAGGGGATGGAG-3' |
| *EMCN* | F' | 5'-CAGCAACCAGCCGGTCTTATTCC-3' |
|  | R' | 5'-GGATCTGCCTTCCAGCACATTCG-3' |
| *CNNM3* | F' | 5'-GACCCCTTCTACGAGGTCCT-3' |
|  | R' | 5'-AAGCAGGCTTCCTCTTCACC-3' |
| *MagT1* | F' | 5'-AGTTCCGTCGCCTTGTGAAA-3' |
|  | R' | 5'-TCAGCTTGCTTGCAAACGAC-3' |
| *EMCN* | F' | 5'-CAGCAACCAGCCGGTCTTATTCC-3' |
|  | R' | 5'-GGATCTGCCTTCCAGCACATTCG-3' |
| *VEGFA* | F' | 5'-AGGAGGAGGGCAGAATCATCACG-3' |
|  | R' | 5'-GGGCACACAGGATGGCTTGAAG-3' |
| *ANG* | F' | 5'-GTTGGTCTTCGTGCTGGGTCTG-3' |
|  | R' | 5'-GTGCTGGGTCAGGAAGTGTGTG-3' |
| *DSPP* | F' | 5'-TGGAGCCACAAACAGAAGCA-3' |
|  | R' | 5'-TCATTGTGACCTGCATCGCC-3' |
| *RUNX2* | F' | 5'-CCCACGACAACCGCACCATG-3' |
|  | R' | 5'-GCAGCACCGAGCACAGGAAG-3' |

**Western blot raw images**


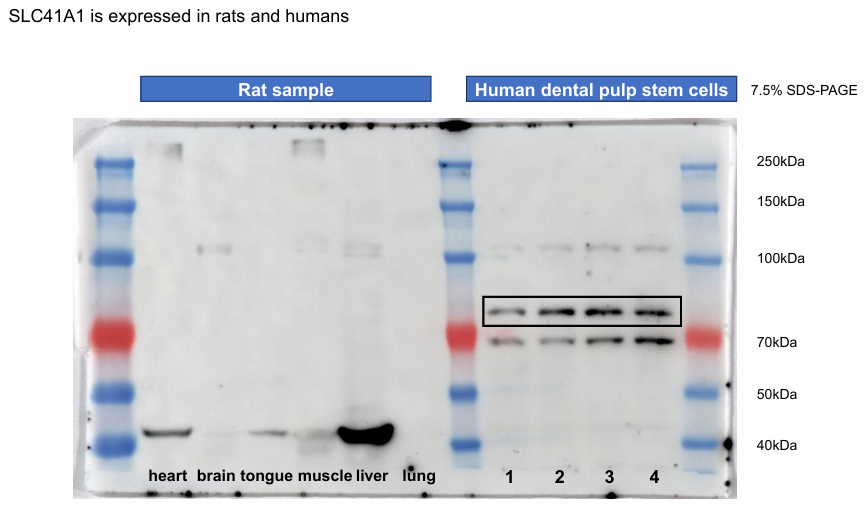


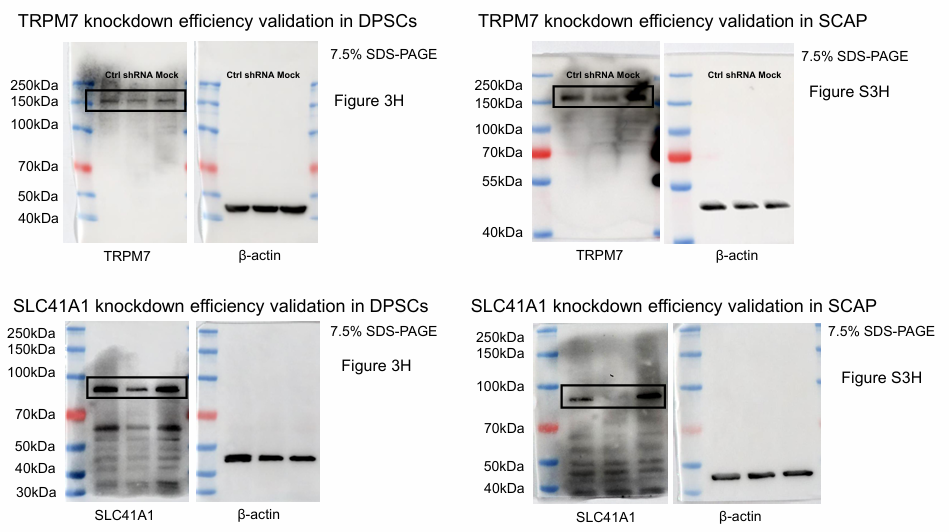


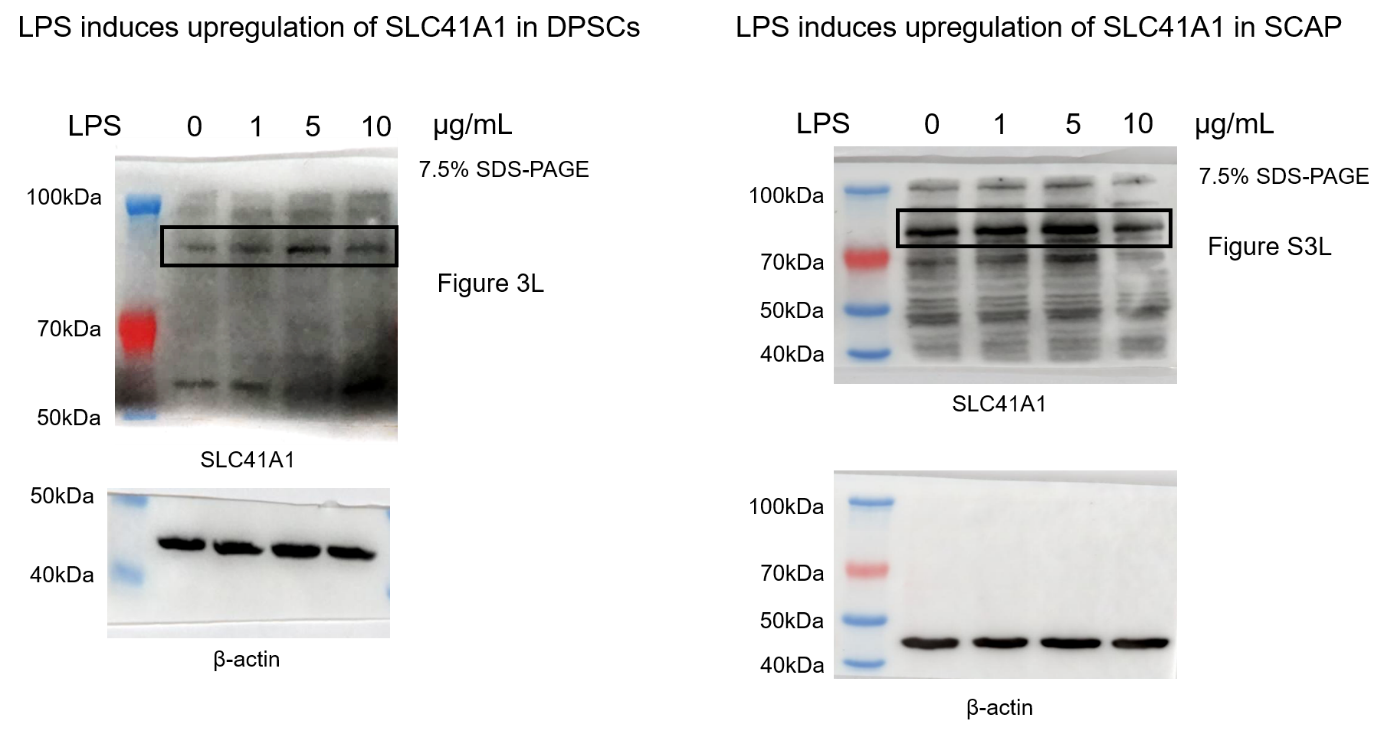


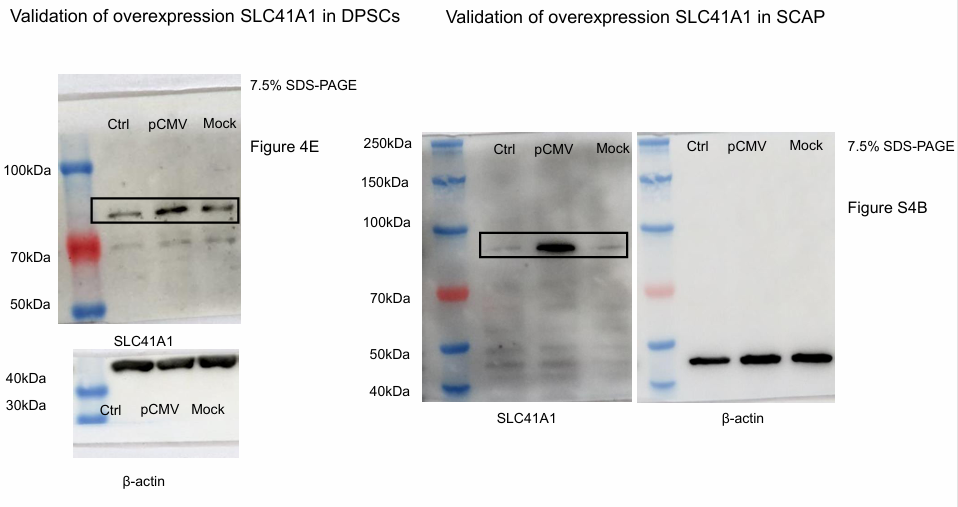


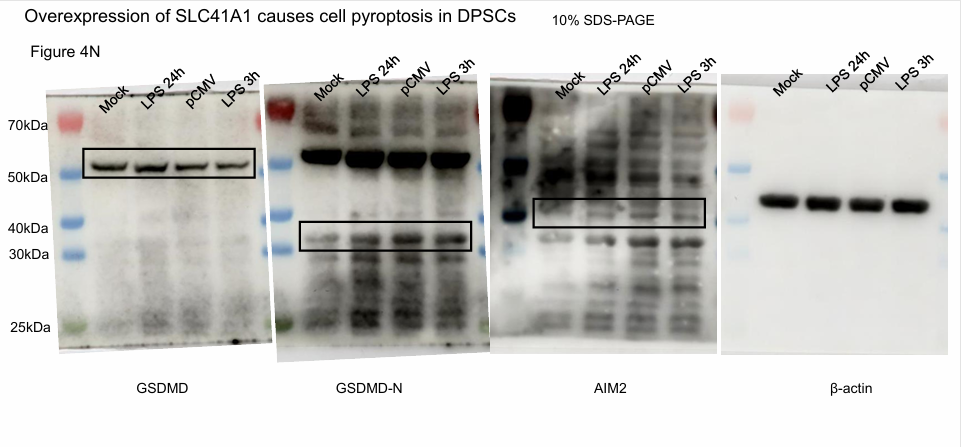

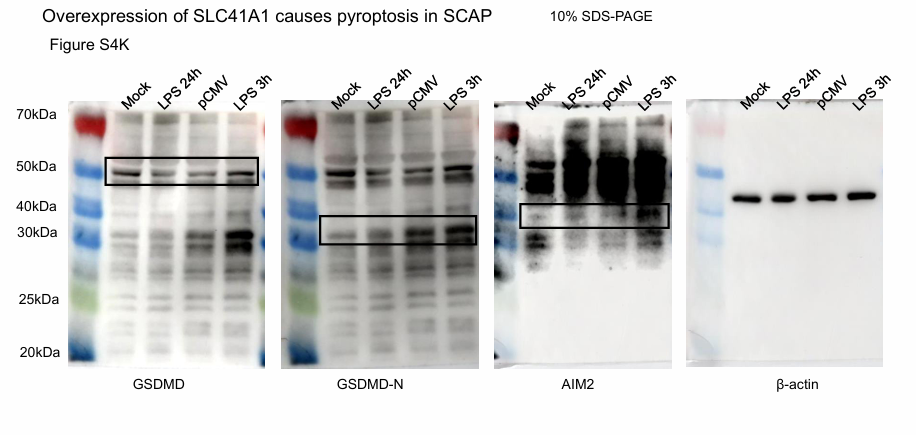


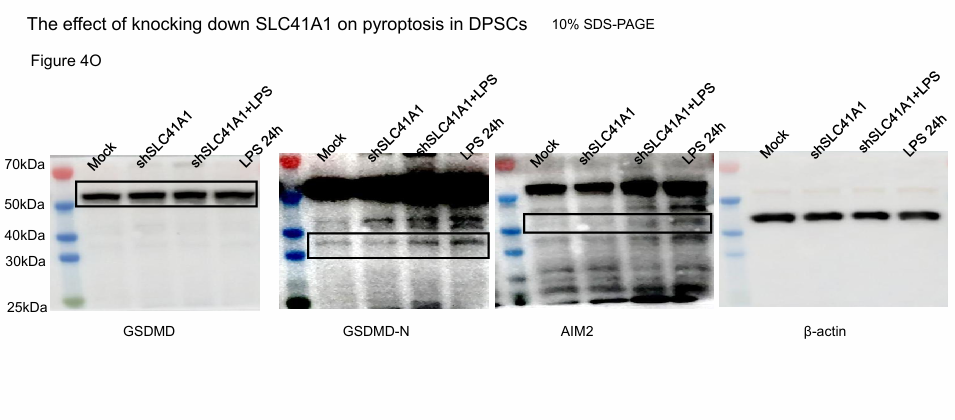


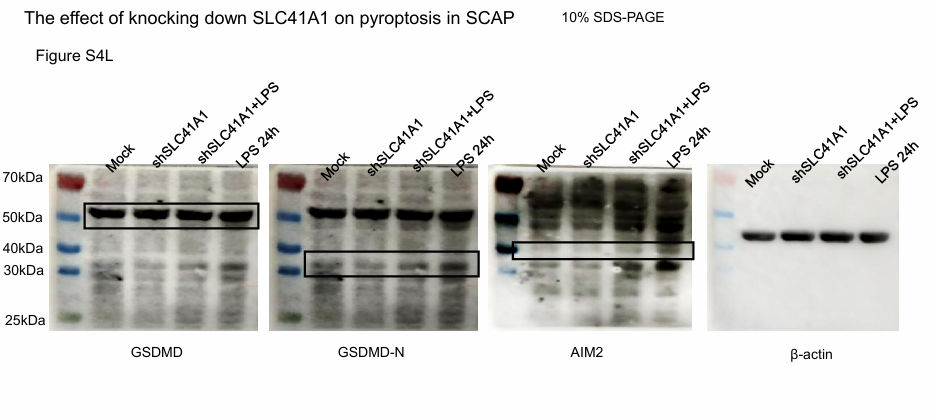


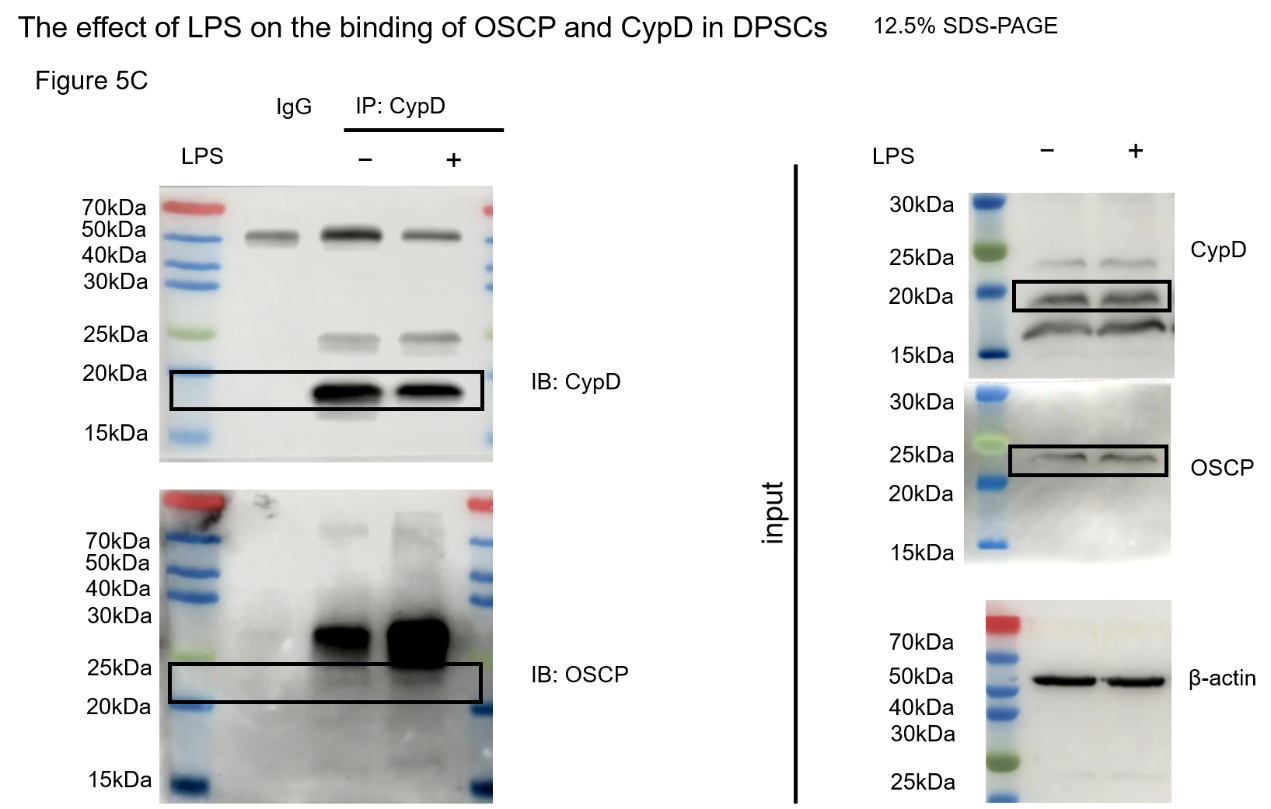


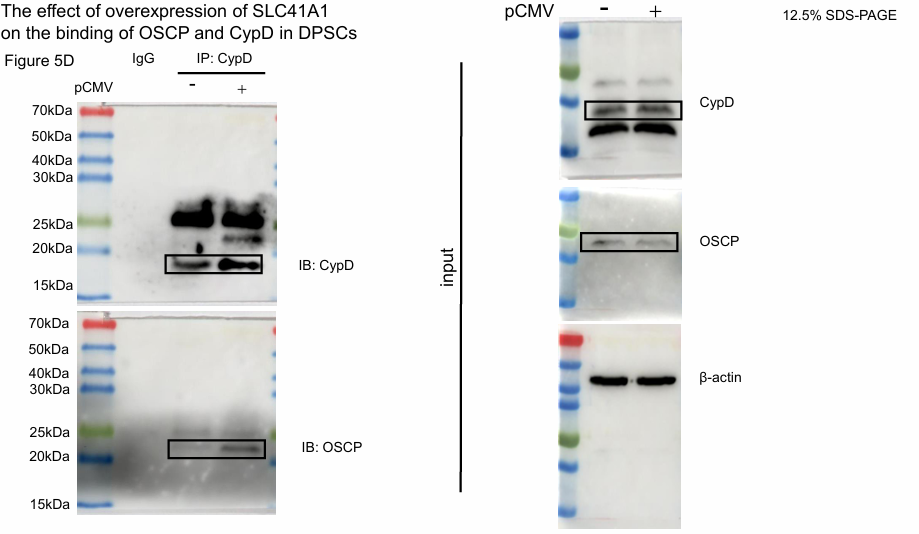


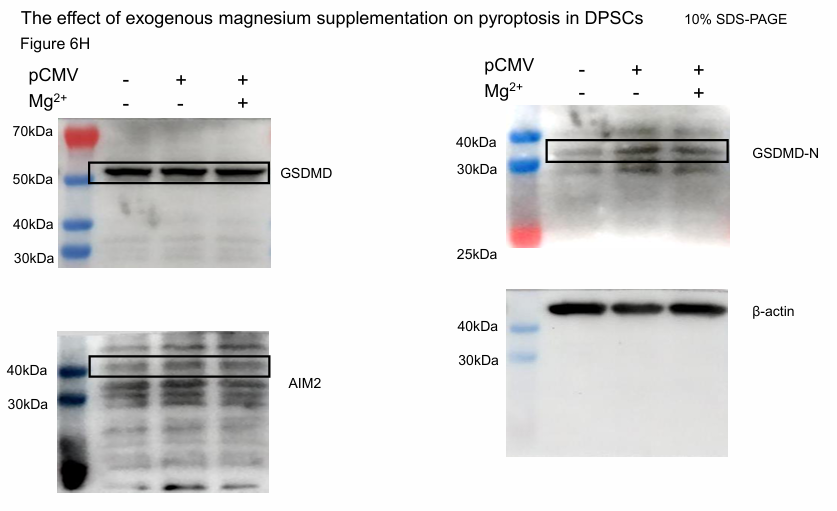


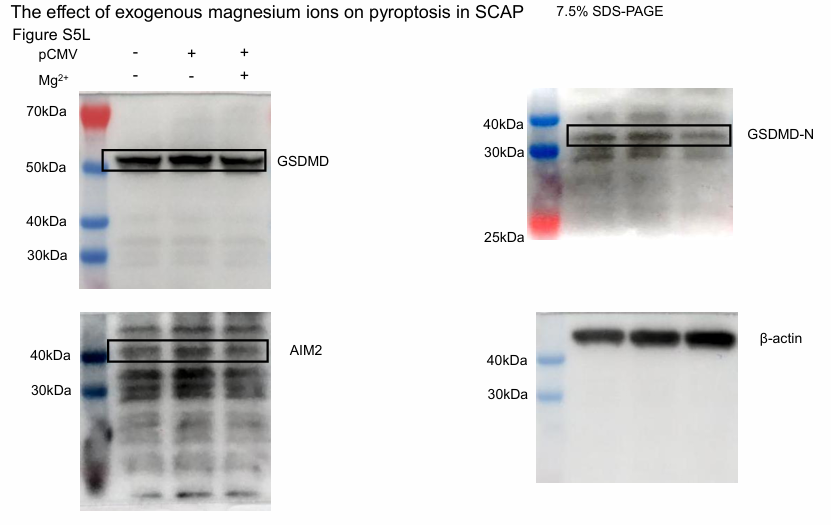


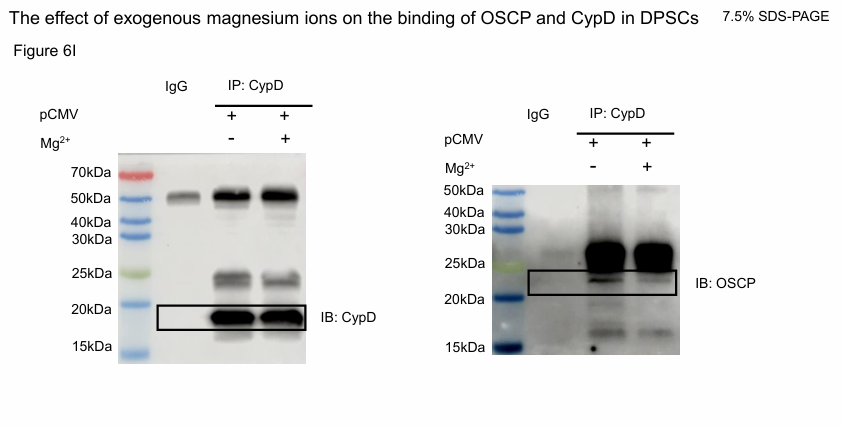


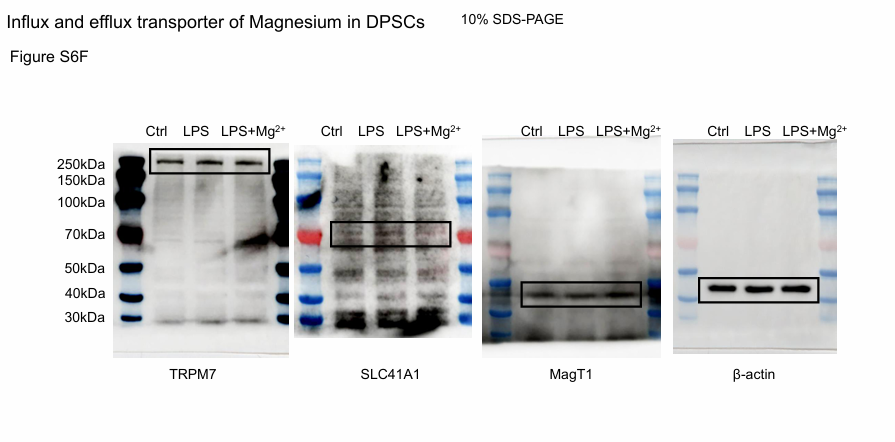

Supplement: Supplementary file 1 — Supporting Information [file ADVS-12-e05666-s001.docx]
